# Novel reactions of a profluorescent nitroxide probe with ozone showcase a cascade of solvent-dependent redox reactions†

**DOI:** 10.1039/d5ra03412d

**Published:** 2025-07-23

**Authors:** Zachary E. Brown, Carl P. Soltau, David L. Marshall, Steven E. Bottle, Branka Miljevic

**Affiliations:** a School of Earth and Atmospheric Sciences, Queensland University of Technology GPO Box 2434 4001 Brisbane Australia b.miljevic@qut.edu.au; b School of Chemistry and Physics, Queensland University of Technology GPO Box 2434 4001 Brisbane Australia c.soltau@qut.edu.au; c Central Analytical Research Facility and School of Chemistry and Physics, Queensland University of Technology GPO Box 2434 4001 Brisbane Australia

## Abstract

The accurate detection of airborne pollutants remains critical for safeguarding both environmental integrity and public health. Equally important is the consideration of method stability and susceptibility to degradation by common reactive atmospheric species, such as ozone. This study examines the reactivity of ozone towards the profluorescent nitroxide (PFN) BPEAnit, a molecular probe that is used in an acellular assay for detecting reactive oxygen species (ROS) from particulate pollution. Online fluorescence measurements revealed a dose-dependent increase in fluorescence when a BPEAnit/DMSO solution was exposed to low-level ozone concentrations (0–544 ppb). Exposure to excess ozone (*ca.* 9.5 ppm) produced sufficient fluorescent products for LC-MS analysis, which, when combined with isotope labelling, enabled structural characterization of several products, accounting for 43.5% of the total fluorescent signal. Under similar conditions the parent fluorophore (BPEA) showed no reactivity toward ozone, confirming the specificity of BPEAnit. The primary mechanism is proposed as a single-electron transfer between BPEAnit and ozone, forming an oxoammonium cation and ozone radical anion, which react with DMSO to yield carbon- and sulfur-based adducts. Increased moisture significantly altered the product distribution, highlighting the need to consider ambient humidity in these atmospheric assays. Preliminary evaluation of alternative solvent systems, ethanol and cyclohexane, revealed simpler reaction profiles with fewer products; however factors such as solvent volatility, ozone dose-response, and product stability require further investigation. These findings support the reliability of the BPEAnit probe towards ozone, as well as demonstrating a sensitive, ozone-responsive fluorescence profile, offering potential for broader application in atmospheric monitoring.

## Abstract 2

This study explores the reactivity of the profluorescent nitroxide BPEAnit with ozone, showing a dose-dependent fluorescence response that yields multiple products influenced by solvent system and moisture content.

## Graphical abstract

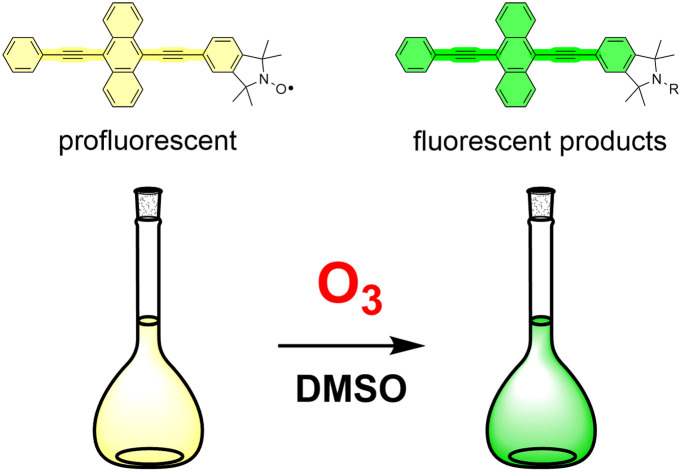

## Introduction

Poor air quality is a rapidly expanding global health concern that is well known to cause several adverse health effects and premature deaths.^1^ Air pollution-induced health effects stem from oxidative stress and inflammation triggered by the inhalation of redox-active pollutants, including carbon monoxide, nitrogen dioxide, ozone, and fine (≤2.5 μm) and ultrafine (≤0.1 μm) particulate matter. These pollutants contain or generate reactive oxygen species (ROS) that disrupt the oxidant-antioxidant balance in the epithelial lining fluid of the respiratory tract and can enter the bloodstream.^2,3^

**Scheme 1:**
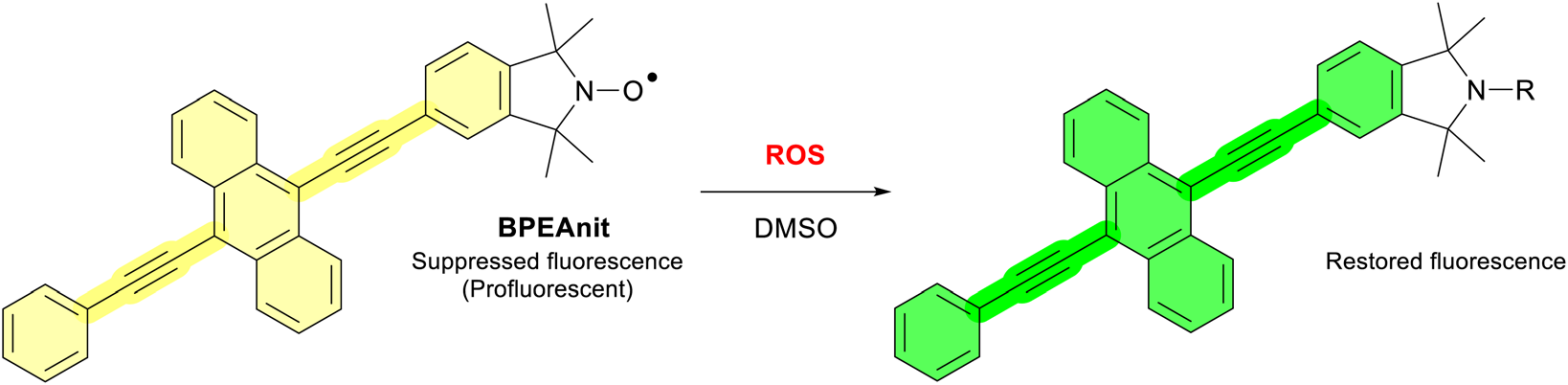
Illustrating the ROS-driven fluorescent response of the PFN BPEAnit when solvated in DMSO.

Due to the highly reactive nature of ROS and difficulties surrounding their isolation, many types of molecular probes have been developed to directly detect and quantify these reactive species. Previously our group has analysed several sources of atmospheric pollution for their ROS composition using the profluorescent nitroxide (PFN) probe BPEAnit.^4–9^ This methodology utilises two unique properties of nitroxides; their efficient radical scavenging activity,^10,11^ and intermolecular fluorescence quenching,^12,13^ to give a ROS sensitive fluorescent response (Scheme 1). The effectiveness of the BPEAnit is owed to its strongly suppressed fluorescence, which once restored can return a signal that is 300-fold higher in intensity.^14^ When using PFNs to detect and quantify free radicals and ROS in pollution sources, it is essential to consider their chemical stability and vulnerability to degradation by common atmospheric pollutants (*i.e.* ozone).

Ozone is a strongly oxidizing gaseous molecule and a type of ROS, formed naturally through a complex series of reactions in the troposphere. Ozone is well known to perform ozonolysis on unsaturated alkene and alkyne functional groups, resulting in the formation of various carbonyl-based groups.^15^ In the case of BPEAnit, these reactions may cause a change in fluorescence intensity due to modification of the fluorophore and thus the emission wavelength, leading to an under or overestimation of ROS concentration. Furthermore, in the lower troposphere ozone is detrimental to human health. Upon inhalation, ozone reacts to form secondary oxidation products that induce intracellular oxidative stress, ultimately leading to apoptosis.^16,17^ It is therefore of interest to investigate not only the stability of BPEAnit towards ozone, but also whether the DMSO-solvated acellular assay can be used to detect and quantify ozone. Ozone can react with atmospherically relevant substrates to generate reactive species (*e.g.*, ·OH) or directly with DMSO,^18^ leading to secondary oxidation products which may interact with BPEAnit and trigger a fluorescence response. This study examines the sampling of ozone with and without the presence of known radical-generating substrates in BPEAnit-containing solutions, aiming to identify any trapped radical adducts and/or degradation products that may form. To achieve this, we employed HPLC coupled with online fluorescence and mass spectrometry to identify fluorescent products that result upon exposure of BPEAnit in DMSO solution with ozone. We found that ozone levels even at atmospherically relevant concentrations (*ca.* ≤100 ppb) caused a noticeable increase in fluorescence. Moreover, at higher concentrations and extended exposure times, we were able to enhance product generation and begin characterising the complex mixture using fluorescence HPLC-MS.

## Experimental

### Chemicals

Dimethyl sulfoxide (99%) was purchased from ChemSupply and used as received. Dimethyl sulfoxide-*d*_6_ (99.9%) was purchased from Novachem and used as received. Cyclohexane (99.5%) and ethanol (99.5%) were purchased from Fischer Scientific and used as recieved. 9,10-Bis(phenylethynyl)anthracene (BPEA) 97% was purchased from Sigma Aldrich and used as received. 9,10-Bis (phenylethynyl) anthracene-nitroxide (BPEAnit) and 1,1,3,3-tetramethylisoindolin-2-yloxyl (TMIO) was provided by Prof. Steven Bottle.^14^

### Analytical

High-performance liquid chromatography was performed using a Dionex UltiMate™ 3000 RSLC with a Restek Ultra C18 column (200 mm × 4.6 mm × 5 μm) maintained at 40 °C. Generally, isocratic elution using 100% Optima™ LC/MS grade acetonitrile (Thermo Fischer, Cat#A9554) was used as the elutant. Fluorescence emission (FLD) intensity (*λ*_ex_ = 434 nm, *λ*_em_ = 492 nm) was measured using the Dionex UltiMate™ 3000 FLD-3000 module. UV absorbance was measured at 254 nm and 434 nm using the UltiMate™ DAD 3000. High-resolution mass spectrometry was performed using a Thermo Fischer Orbitrap Elite hybrid ion trap mass spectrometer operated in positive ion mode at a mass resolution of 240 000 for masses 200–2000 *m*/*z*.

### Ozone generation

Ozone was generated using a dry 80 : 20 mix of ultra-high purity nitrogen and oxygen that was passed through an ozone generator (Analytik Jena, USA). Different ozone concentrations were achieved by adjusting the lamp sheath of the 185 nm lamp in the ozone generator. The gas flow rate through the ozone generator was 2 L min^−1^ and split into two paths (Fig. S2†). One path went to an Ecotec EC9810 ozone analyser that was sampling at a flow rate of 0.5 L min^−1^, and the remaining flow into the impinger was 1.5 L min^−1^. N_2_ and O_2_ flow rates were controlled using mass flow controllers (Alicat Scientific). All tubing connections used Swagelok fittings attached to Teflon tubing.

### Ozone sampling

The sampling method was adapted from our previously reported methodology.^5^ Test compounds were first dissolved in the appropriate solvent to the desired concentration before being quantitatively transferred into the impinger vessel. The desired concentration of ozone was introduced through a custom-made impinger^19^ with a fritted nozzle tip into a gas-tight vessel containing the analyte solution (typically 20 mL) at a rate of 1.5 L min^−1^, with liquid flow ports at the bottom of the vessel to allow for continuous liquid circulation through a fluorometer (Fig. S1†). Sampling was performed for the desired time frame, with 0.5 mL aliquots taken for LC-MS analysis.

### Continuous fluorescence emission monitoring

Fluorescence measurements were taken continuously at 492 nm by an Ocean Optics USB2000+ spectrometer excitation by 450 nm Thorlabs diode laser with a custom-designed flow-through cell.^20^ The liquid flow was controlled by an Ismatec Reglo ICC peristaltic pump to give a constant liquid flow rate of 1 mL min^−1^.

### Coulometric titration

The water content of DMSO was analysed by using a Mettler Toledo C10s Coulometric KF titrator using Hydranal™ Coulomat AG. The mass of the sample added was entered and the resulting water content output in ppm.

## Results and discussion

### Reaction of BPEAnit and BPEA in DMSO with ozone

**Fig. 1:**
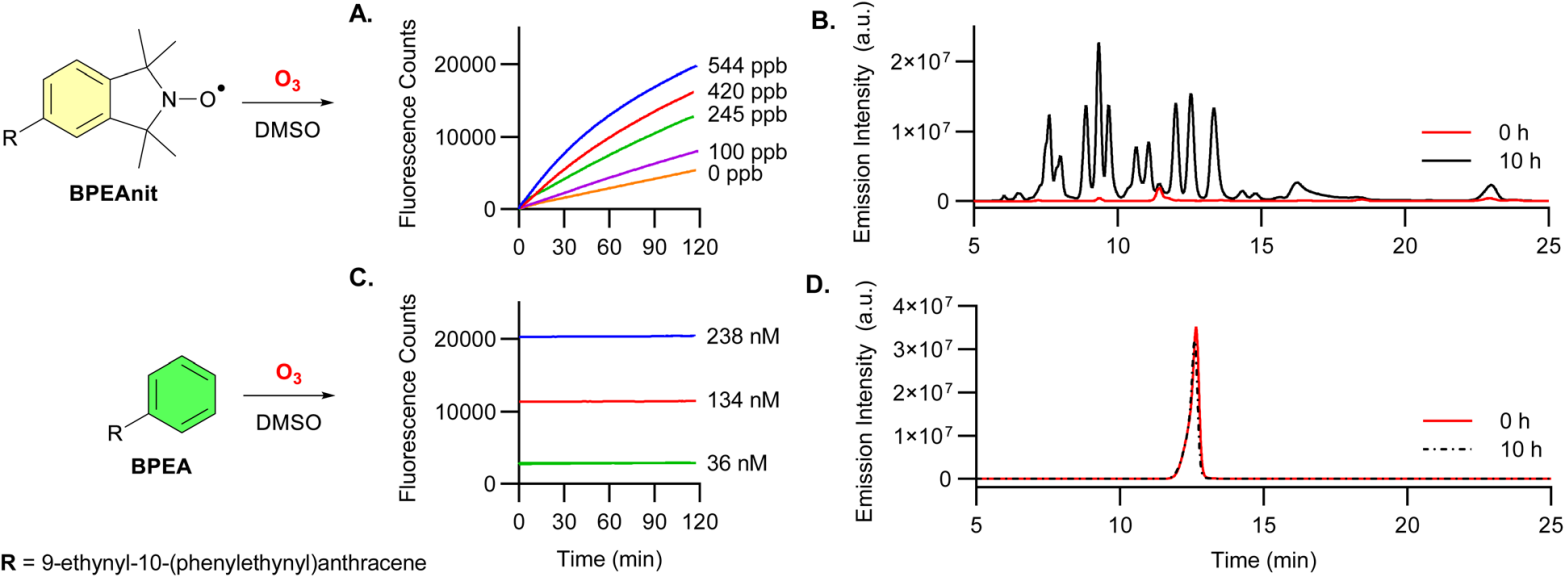
(A) Continuous online fluorescence of 4 μM BPEAnit in DMSO during exposure to a constant stream of various ozone concentrations at 1.5 L min^−1^. Fluorescence HPLC trace (*λ*_ex_ = 434 nm, *λ*_em_ = 492 nm) of DMSO solutions containing 0.5 mM of BPEAnit (B) or parent fluorophore BPEA (D) before (0 h) and after (10 h) exposure to a constant stream of ozone (*ca.* 9.5 ppm) at 1.5 L min^−1^. (C) Continuous online fluorescence of varying concentrations of BPEA in DMSO when exposed to a constant stream of ozone (544 ppb) at 1.5 L min^−1^.

Varying concentrations of generated ozone in the range of 0–544 ppb were drawn through a solution of BPEAnit (4 μM) in DMSO at a constant rate of 1.5 L min^−1^ using an impinger, as previously described.^19^ Through continuous online fluorescence emission monitoring, we observed an increase in fluorescence (*λ*_ex_ = 434 nm, *λ*_em_ = 492 nm) over time in a dose-dependent manner (Fig. 1A). This demonstrates that at a concentration of 4 μM in DMSO, BPEAnit produces a measurable fluorescence response to ozone concentrations of ≥100 ppb. Notably, this detection threshold aligns with the globally recognised permissible exposure limit for occupational ozone exposure.^21^ To determine whether this response is driven by nitroxide radical scavenging or modification of the fluorophore—particularly *via* ozonolysis of the alkyne groups—we exposed the parent fluorophore (BPEA) to similar conditions. No change in fluorescence was observed after 2 hours (Fig. 1C), indicating that the response is specific to the BPEAnit probe. Notably, a gradual increase in fluorescence for BPEAnit is observed in the absence of ozone, which is likely due to autooxidation.

To obtain deeper insight of the fluorescence response we sought to maximise product generation and enable subsequent analysis by LC-MS. To achieve this the concentration of BPEAnit was increased to 0.5 mM and exposed to a large excess of ozone (*ca.* 9.5 ppm) for 10 hours. This resulted in a substantial increase in product formation as observed by fluorescence HPLC (Fig. 1B and S7†). BPEA was exposed to the same elevated ozone concentration to assess for any degradation, though no changes were observed over the duration of the experiment (Fig. 1D and S4†). These findings indicate that structure of BPEA remains unchanged even after prolonged exposure to a ∼20-fold excess of ozone, suggesting that the fluorophore of BPEAnit is unaffected by ozone under these conditions. Given that the ozone concentrations used far exceeds ambient levels and no fluorophore-modifying reactions were observed, it is unlikely that atmospheric ozone concentrations would interfere with the performance of BPEAnit in the acellular assay. Consequently, the fluorescence response observed upon exposure of the BPEAnit-DMSO system to both atmospherically relevant and elevated ozone concentrations can therefore be attributed specifically to its reaction with ozone.

### Characterising the fluorescence response

**Fig. 2:**
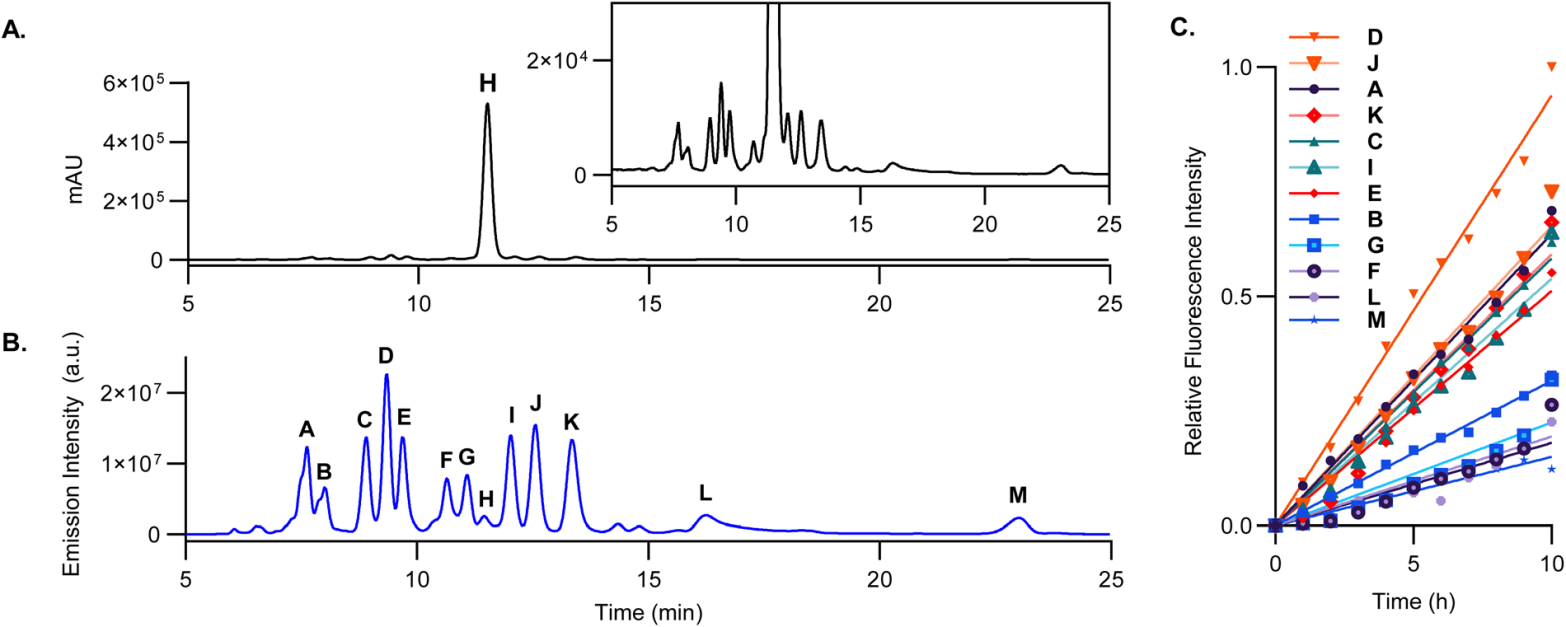
UV HPLC-MS chromatogram (A) and fluorescence HPLC–MS trace (*λ*_ex_ = 434 nm, *λ*_em_ = 492 nm) (B) of 0.5 mM BPEAnit in DMSO after 10 h exposure to a constant stream of ozone (*ca.* 9.5 ppm) at 1.5 L min^−1^. Peaks of significance are labelled A–M, where H is BPEAnit. Relative change in HPLC fluorescence intensity peak area over 10 hours (C), with data normalised to the highest observed signal (peak D, 1.37 × 10^8^ a.u.), where the figure legend is arranged in descending order of fluorescence increase over time. A simple linear regression was applied using GraphPad Prism 10.4.1.

Despite minimal detection by standard UV-vis HPLC (Fig. 2A), the fluorescence HPLC trace of BPEAnit after 10 hours of excess ozone exposure reveals the formation of several fluorescent products, labelled A–M (Fig. 2B). Hourly sampling throughout the exposure period indicates that the individual fluorescence signals from these products emerge at relatively constant rates, with peak D being the most significant (Fig. 2C and S7†). The observed mass-to-charge ratios and their closest assigned molecular formulas are compiled in Table 1. Previous studies by Stevanovic *et al.* and Soltau *et al.* have collectively isolated and characterised several products resulting from radical reactions with DMSO in the presence of BPEAnit or the parent nitroxide.^5,22^ By comparing these findings with the results of this study, we are able to identify several of the fluorescent compounds contributing to the overall fluorescence response (Table 2). Several peaks correspond to products formed through the trapping of DMSO-derived radicals by the nitroxide functionality. Based on their high-resolution mass, peaks C and D are assigned the structures of compound 2 and 3, respectively, indicating the trapping of the sulfur-centred radicals ·SOCH_3_ and ·SO_2_CH_3_. Additionally, peak L and M aligned with compounds 5 and 6, suggesting the capture of the carbon-centred radicals ·CH_2_SOCH_3_ and ·CH_3_. Furthermore, peaks B and E are assigned based on accurate mass to the odd nitroxide fragmentation compounds 1 and 6.

**Table 1:** Retention time (*t*_R_), % of total fluorescence and observed mass-to-charge ratio (ESI) for peaks A–M from Fig. 2. Closest assigned molecular formula were assigned using the observed high-res *m*/*z* in the elemental composition tool in Xcalibur 3.0

Peak	*t*_R_ (min)	FLD (%)	Observed *m*/*z*	Closest assigned molecular formula	Δ*m*/*z* (ppm)
A^a^	7.6	9.4	539.2084	C_36_H_29_NO_4_ (539.2086)	0.37
B	8.0	4.6	504.1953	C_36_H_26_NO_2_ (504.1958)	0.99
C	8.9	10.3	569.2012	C_37_H_31_NO_3_S (569.2019)	1.23
D	9.3	17.4	553.2066	C_37_H_31_NO_2_S (553.2070)	0.72
E	9.6	9.9	476.2006	C_35_H_26_NO (476.2009)	0.63
F^a^	10.6	4.7	505.2039	C_36_H_27_NO_2_ (505.2042)	0.59
G^c^	11.0	5.5	—	—	—
H^b^	11.4	1.1	490.2165	C_36_H_28_NO (490.2165)	0
I^c^	12.0	10.1	—	—	—
J^c^	12.5	11.0	—	—	—
K	13.3	8.9	504.2318	C_37_H_30_NO (504.2322)	0.79
L	16.2	1.2	568.2302	C_38_H_34_NO_2_S (568.2305)	0.53
M	23.0	0.1	506.2476	C_37_H_32_NO (506.2478)	0.40

a Observed *m*/*z* did not match any previously identified compounds.

b Parent compound BPEAnit.

c Molecular ion could not be assigned. FLD (%) represents the proportion of the area under the curve for a specific peak relative to the total area under the curve for all detected peaks.

**Table 2:** Comparison of high-resolution MS (ESI) *m*/*z* peaks after a 10-hour exposure of a 0.5 mM BPEAnit solution in DMSO or DMSO-*d*_6_ to a constant ozone stream (*ca.* 9.5 ppm) at 1.5 L min^−1^. Molecular assignments are based off findings from prior studies by Stevanovic *et al.*^5^ and Soltau *et al.*^22^ Deuterated hydrogen atoms for DMSO-*d*_6_ are highlighted

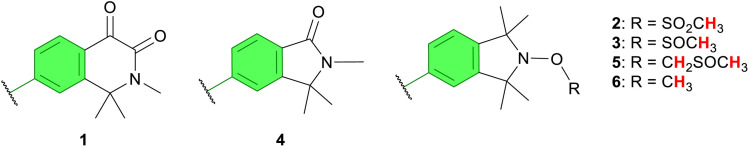					
Peak	DMSO		DMSO-*d*_6_		Assignment
	Observed *m*/*z*	Expected *m*/*z*	Observed *m*/*z*	Expected *m*/*z*	
B	504.1953 [M + H]^+^	504.1958 [M + H]^+^	No change	No change	1
C	569.2012 [M]^+^	569.2025 [M]^+^	572.2200 [M]^+^	572.2213 [M]^+^	2
D	553.2066 [M]^+^	553.2075 [M]^+^	556.2234 [M]^+^	556.2264 [M]^+^	3
E	476.2006 [M + H]^+^	476.2009 [M + H]^+^	No change	No change	4
L	568.2302 [M + H]^+^	568.2305 [M + H]^+^	573.2616 [M + H]^+^	573.2619 [M + H]^+^	5
M	506.2476 [M + H]^+^	506.2478 [M + H]^+^	509.2661 [M + H]^+^	509.2667 [M + H]^+^	6

To further validate these assignments, we conducted an additional ozone sampling reaction, replacing DMSO with DMSO-*d*_6_ as the solvent. The resulting solution was then analysed by LC-MS to detect mass shifts corresponding to the expected number of deuterium atoms. Upon examining the LC-MS data, deuterated signals were observed for compounds 2, 3, 5, and 6, as expected, but no such shifts were detected for compounds 1 and 4 (Table 2). Through these assignments, 43.5% of the total fluorescence response has been structurally identified. The primary fluorescent species generated from the reaction, accounting for 17.4% of the total fluorescence is the methanesulfonamide adduct (compound 3). This result aligns with the findings reported by Stevanovic *et al.* where compound 3 was also the main fluorescent species generated.^5^ To determine whether similar reactions occur in the absence of the fluorophore, the parent nitroxide compound 1,1,3,3-tetramethylisoindolin-2-yloxyl (TMIO) was dissolved in DMSO and exposed to excess ozone (*ca.* 9.5 ppm). This resulted in the formation of multiple distinct peaks by LC–MS, with several exhibiting equivalent mass-to-charge values comparable to those observed in the previous study by Soltau *et al.* (Fig. S23 and Table S1†).^22^ These results clearly demonstrate that sampling ozone through the DMSO solution of BPEAnit induces radical formation and subsequent nitroxide scavenging, leading to a rapid fluorescence turn-on of BPEAnit.

### Proposed mechanism

**Scheme 2:**
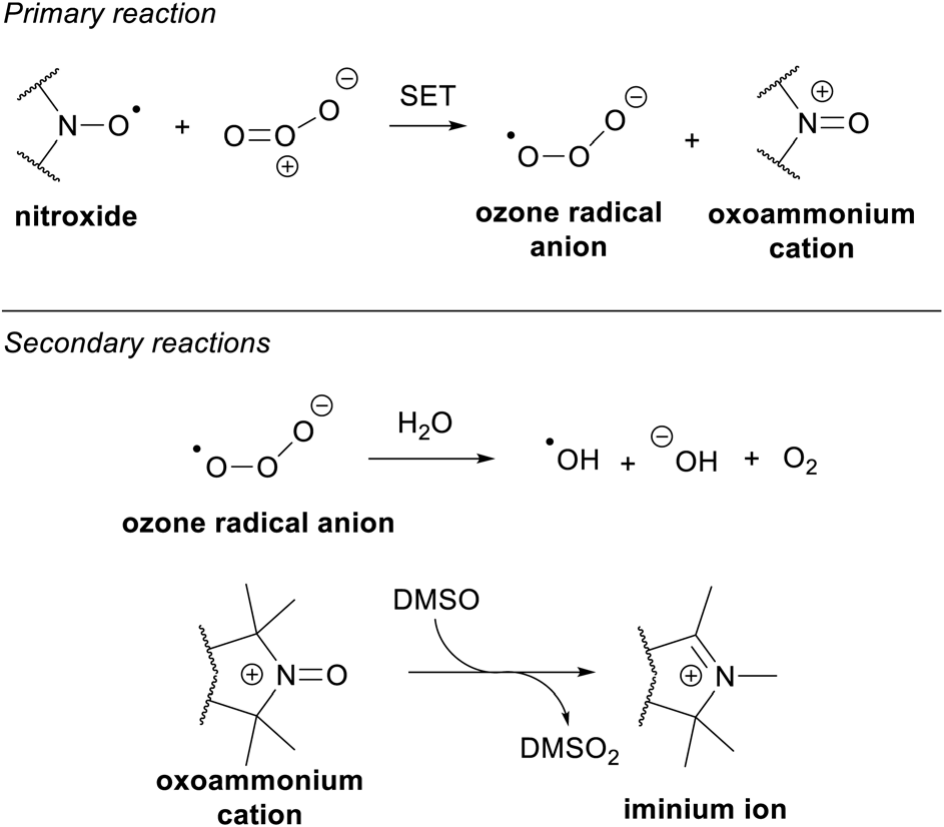
Proposed mechanism of the primary and secondary reactions resulting from the reaction of BPEAnit with ozone in DMSO.

Based on the components we have identified, we propose the following mechanism for the generation of the products and, consequently, the fluorescence response (Scheme 2). As ozone is known to readily participate in single-electron transfer (SET) reactions,^23,24^ we suggest that the primary reaction involves the oxidation of BPEAnit to the oxoammonium cation, accompanied by the generation of the ozone radical anion. Since the redox potential of the O_3_/O_3_˙^−^ couple (+1.6 V)^23,24^ is significantly higher than that of the oxidation potential of isoindoline nitroxides (+0.7–0.9 V),^25^ the proposed reaction of nitroxide with ozone is thermodynamically favourable. The ozone radical anion may then react with adventitious water, creating hydroxide ion, diatomic oxygen, and the highly-reactive hydroxyl radical,^26^ which can further react with DMSO to produce various radical species.^22,27–29^ The oxoammonium cation can undergo an oxygen atom transfer reaction with DMSO, leading to N–O bond cleavage and methyl migration to form an iminium ion,^30^ which in the presence of oxidants is reported to yield lactam 4 and oxo-lactam 6.^22^

### Effect of water concentration

**Fig. 3:**
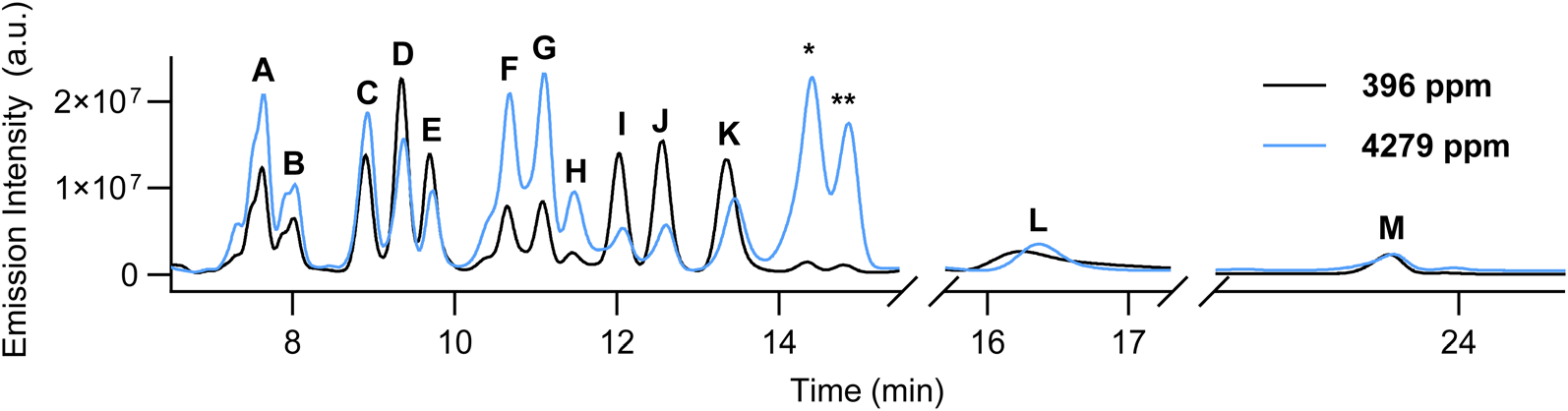
Fluorescence HPLC trace (*λ*_ex_ = 434 nm, *λ*_em_ = 492 nm) demonstrating the effect of water concentration (396 ppm H_2_O & 4279 ppm H_2_O) on the product generation of 0.5 mM BPEAnit in DMSO after 10 h exposure to a constant stream of ozone (*ca.* 9.5 ppm) at 1.5 L min^−1^.

As water is the proposed substrate to generate the highly-reactive hydroxyl radical, we sought to investigate how water concentration influences product formation. Although water was never intentionally added to the system, moisture from the air inevitably diffuses into the solution over time due to the hygroscopic nature of DMSO.^31^ Coulometric titration determined the water content of the starting DMSO solution to be 396 ppm, which is comparable to water concentrations found in relatively dry air conditions (≤500 ppm, approximately 2% relative humidity at 25 °C). To investigate the impact of humidity variations on this sampling method, we adjusted the water content of the DMSO starting solution to a titrated concentration of 4279 ppm and repeated the 10-hour ozone sampling experiment. Upon LC-MS analysis we observed no new products, however there was a substantial change in the ratio of products (Fig. 3, Tables S2 and S3†). Peak integration indicated that peaks A–C and K–L remained relatively unchanged, with the largest variation being a 3.6% reduction in area for peak K. In contrast, peaks D–G, I, and J exhibited significant changes, with area variations ranging from a 4.9% to an 11.3% shift. Notably, two previously minor products, denoted as “*” and “**”, emerged as the dominant fluorescent species in this instance, increasing from <0.1% to 14.1% and 8.9%, respectively. Attempts to characterise these compounds were unsuccessful, as their observed *m*/*z* of 602.1099 did not correspond to any plausible structure or molecular formula. These findings suggest that humidity can strongly influence the chemical composition of the fluorescent compounds, especially considering that typical ambient humidity is ≥40%. A potential strategy to mitigate the impact of moisture on this assay format is the introduction of an inline desiccant (*e.g.* silica gel or calcium chloride). However, the possibility that these materials may scavenge airborne reactive species, and thereby compromise the accuracy of the fluorescence response, must be carefully evaluated.

### Alternative solvent screening

**Fig. 4:**
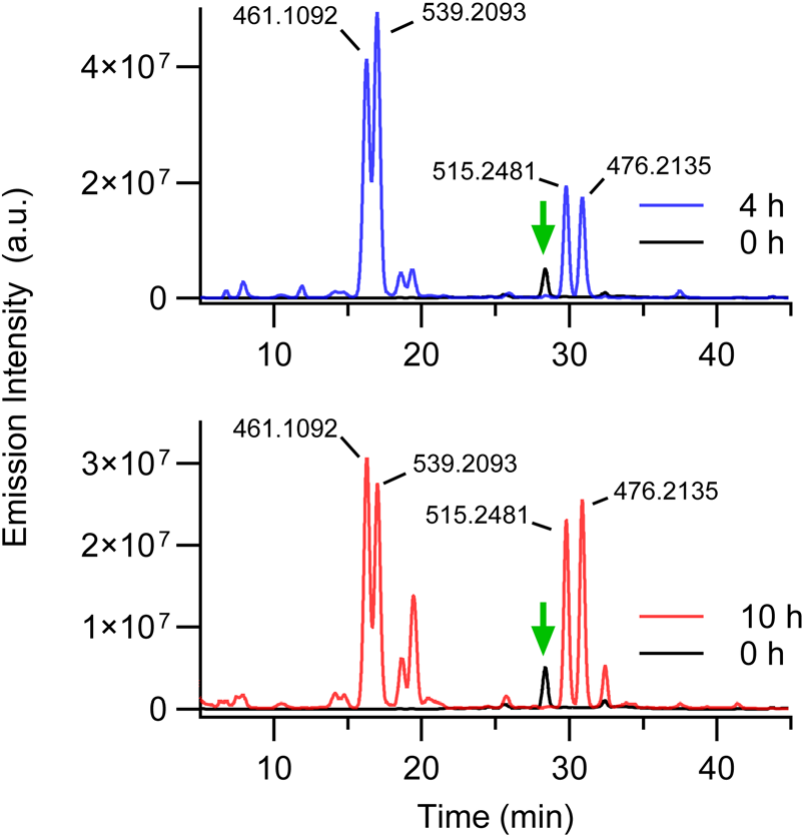
Fluorescence HPLC trace (*λ*_ex_ = 434 nm, *λ*_em_ = 492 nm) of 0.5 mM BPEAnit in ethanol (top) and cyclohexane (bottom) after exposure to a constant stream of ozone (*ca.* 9.5 ppm) at 1.5 L min^−1^. BPEAnit is highlighted by the green arrow. Corresponding *m*/*z* of major products are presented.

Given the complexity of the BPEAnit/DMSO reaction with ozone, we also conducted a brief screening of alternative common lab solvents to assess whether these in-combination with BPEAnit could also provide a fluorescence response to ozone. Ethanol and cyclohexane were selected due to their availability, significantly lower reactivity compared to DMSO, and their ability to solubilise BPEAnit at appropriate concentrations. Furthermore ethanol is less hygroscopic than DMSO, while cyclohexane being non-polar does not exhibit any significant hygroscopic behaviour. Similarly to previous experiments, solutions of BPEAnit (0.5 mM) in ethanol and cyclohexane were exposed to a continuous stream of excess ozone and analysed by LC-MS. Both reactions produced similar profiles, resulting in the generation of several fluorescent products (Fig. 4, Tables S4 and S5†). Notably, the ethanol sampling seemed to produce a fluorescence response at a much faster rate than cyclohexane and DMSO, and was halted after only 4 hours exposure due to the solution becoming visibly intensely fluorescent.

Compared to DMSO, the HPLC traces of the ethanol- and cyclohexane-solvated experiments were significantly simpler, with more distinct major products and fewer overall components. The reduction in product complexity could facilitate a more straightforward analysis of the mixture and minimise the number of compounds requiring characterisation. However, a key limitation of these solvents in this application is their volatility, as samples required small volumes to be replenished every 30 minutes to maintain consistency. This issue was not observed with DMSO, with solvent levels remaining relatively consistent over 10 hours of sampling. To validate their use, further testing across different ozone concentrations would be necessary to identify if they provide a dose-dependent response. Furthermore, DMSO's role as a mediator between reactive species and the resulting BPEAnit adducts is well-established. Therefore, it is essential to investigate the products and underlying mechanisms involved with cyclohexane and ethanol to understand their influence on the reaction outcome. Notably, the observed similarities in their resulting reaction profiles suggest that similar mechanisms may are likely under these solvent conditions. Nonetheless, these findings suggest that the BPEAnit sampling method could be adapted to an alternative solvent system, potentially yielding a narrower range of fluorescent products and minimizing external influences such as humidity on product distribution.

## Conclusions

We conducted a series of experiments to evaluate the fluorescence response of the BPEAnit/DMSO acellular assay upon ozone exposure. When bubbled through a solution of BPEAnit in DMSO, continuous online fluorescence measurements revealed a dose-dependent increase in fluorescence across a range of low-level ozone concentrations (0–550 ppb). The stability of the parent fluorophore BPEA was evaluated for ozone-induced degradation, particularly alkyne ozonolysis, however no such reactivity was observed. To facilitate product characterisation, reactions were also performed under excess ozone conditions (*ca.* 9.5 ppm), leading to an enhanced generation of fluorescent products. LC-MS analysis, combined with isotope labeling, enabled the identification of several fluorescent compounds, collectively accounting for 43.5% of the total fluorescence signal. Based on the identified products, we propose that the primary reaction initiating this cascade of redox processes is a single-electron transfer between BPEAnit and ozone, generating the corresponding oxoammonium cation and ozone radical anion, which then undergo further redox transformations. Moisture content was found to significantly influence product distribution, indicating that humidity should be considered when analysing atmospheric samples. A preliminary screening of alternative solvents, ethanol and cyclohexane, suggested cleaner reaction profiles with fewer overall products; however, factors such as solvent volatility, ozone dose–response, and the stability of resulting products remain to be investigated. In summary, these findings demonstrate that the BPEAnit/DMSO system is not prone to degradation by ozone but instead results in the dose-dependent generation of multiple fluorescent products.

## Author contributions

Zachary Brown: conceptualisation, data curation, formyl analysis, investigation, methodology, validation, visualisation, writing – original draft. Carl Soltau: conceptualisation, data curation, formyl analysis, investigation, methodology, validation, visualisation, writing – original draft, project administration, supervision. David Marshall: data curation, formyl analysis, investigation, methodology, writing – review & editing. Steven Bottle: conceptualisation, project administration, resources, supervision, writing – review & editing. Branka Miljevic: conceptualisation, methodology, project administration, resources, supervision, writing – review & editing.

## Conflicts of interest

There are no conflicts to declare.

## Supplementary Material

RA-015-D5RA03412D-s001

## Data Availability

The data supporting this article have been included as part of the ESI.†
