# Supplementary material for: Novel reactions of a profluorescent nitroxide probe with ozone showcase a cascade of solvent-dependent redox reactions
Source: RSC Adv. 2025 Jul 23;15(32):26395–402. doi: 10.1039/d5ra03412d (PMC12284753; doi:10.1039/d5ra03412d)
Supplement: RA-015-D5RA03412D-s001 [file RA-015-D5RA03412D-s001.pdf]

Supplementary Information (SI) for RSC Advances.
This journal is © The Royal Society of Chemistry 2025

Novel Reactions of a Profluorescent Nitroxide Probe with Ozone Showcase a Cascade of Redox Reactions

Zachary E. Brown,[a]# Carl P. Soltau,[b]# David L. Marshall,[c] Steven E. Bottle[b] and Branka Miljevic[a]*

[a] School of Earth and Atmospheric Sciences, Queensland University of Technology, GPO Box 2434, 4001 Brisbane, Australia. E-mail: b.miljevic@qut.edu.au.

[b] School of Chemistry and Physics, Queensland University of Technology, GPO Box 2434, 4001 Brisbane, Australia. E-mail.: c.soltau@qut.edu.au.

[c] Central Analytical Research Facility and School of Chemistry and Physics, Queensland University of Technology, GPO Box 2434, 4001 Brisbane, Australia.

Supporting Information

Contents

Continuous Fluorescence Emission Monitoring 2
Ozone Generation and Sampling 2
Impinger Collection Efficiency....3
BPEA in DMSO Reaction with Ozone LC-MS chromatograms 4
BPEAnit in DMSO Reaction with Ozone Hourly Sample Chromatograms 6
DMSO Reaction with Ozone Hourly Sample Chromatograms 8
Molecular Ion Identification and Mass Spectra for BPEAnit-derived Products....10
TMIO (Parent Nitroxide) Reaction with Ozone 21
BPEAnit in DMSO Reaction with Ozone (Increased Water Content)....23
BPEAnit in Ethanol Reaction with Ozone 25
BPEAnit in Cyclohexane Reaction with Ozone 26

Continuous Fluorescence Emission Monitoring

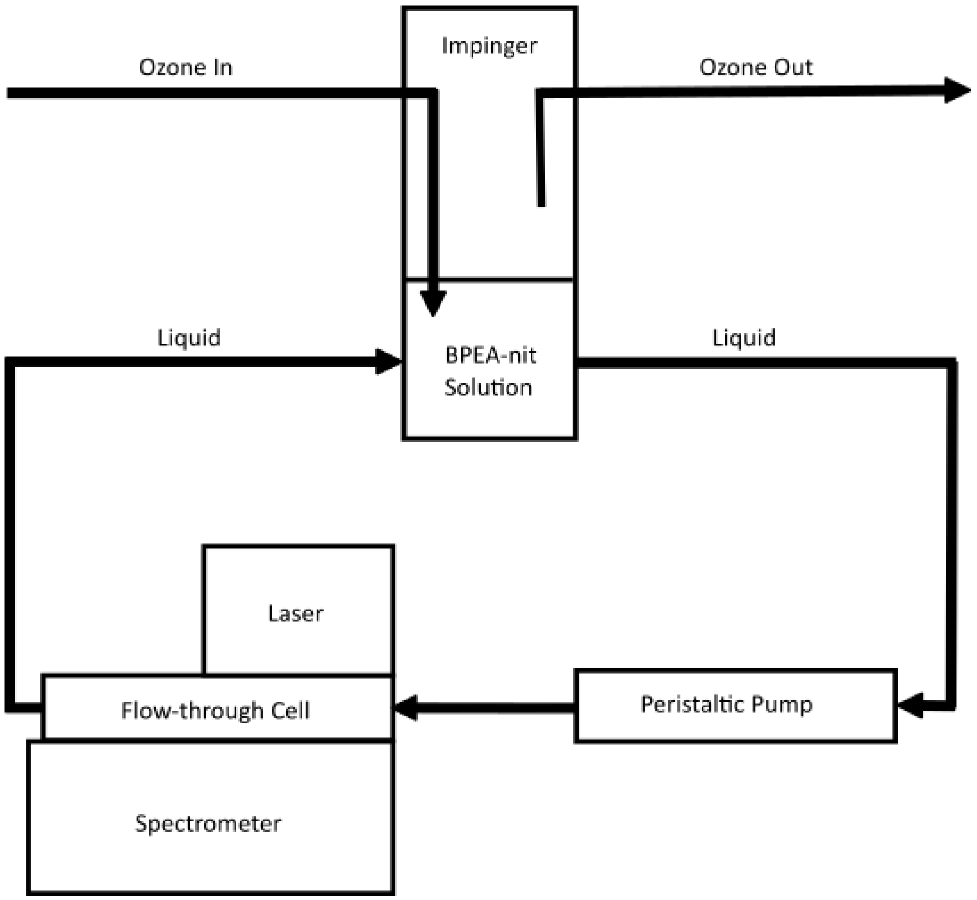

Supplementary Figure 1: Schematic diagram showing the setup for impinger sampling with continuous fluorescence emission measurement at 492 nm.

Ozone Generation and Sampling

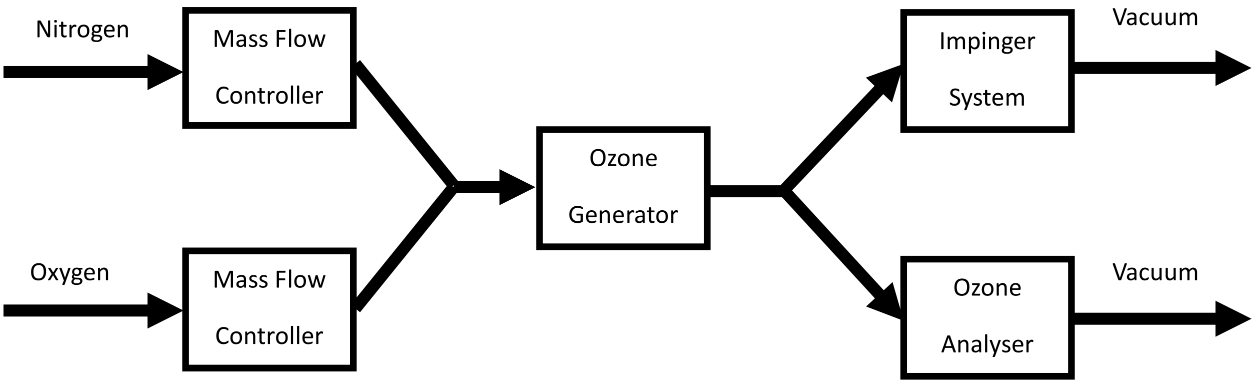

Supplementary Figure 2: Schematic diagram of the experimental setup for generating ozone and sampling into the impinger based system shown in Supplementary Figure 2. Mass flow controllers were used to ensure a constant 80:20 mix of oxygen and nitrogen. The ozone concentration was adjusted by adjusting the lamp sheath of the ozone generator with the resulting concentration being continuously measured by the ozone analyser.

Impinger Collection Efficiency

The ozone collection efficiency for the impingers was completed by measuring the ozone concentration in the gas stream before and after passing through the impinger. This was achieved by using a three-way valve to switch between two gas flow paths manually. An ozone concentration of 500 ppb was used for this experiment. A 20 mL solution of deionised water was used in place of DMSO due to the concern of DMSO vapours damaging the ozone analyser. The flow path was switched every twenty minutes over a period of three hours. This experiment was done in replicate with replicate 1 having a collection efficiency of 19.7 % ± 2.1 and replicate 2 21.0 % ± 3.9. Collection efficiency was calculated by taking mean of the ozone data points after each switch with outliers immediately preceding each switch being removed. Ratios of the concentration of the concentration of ozone before and after the impinger was calculated.

The error for each data point was taken from the standard deviation of the averaging periods.

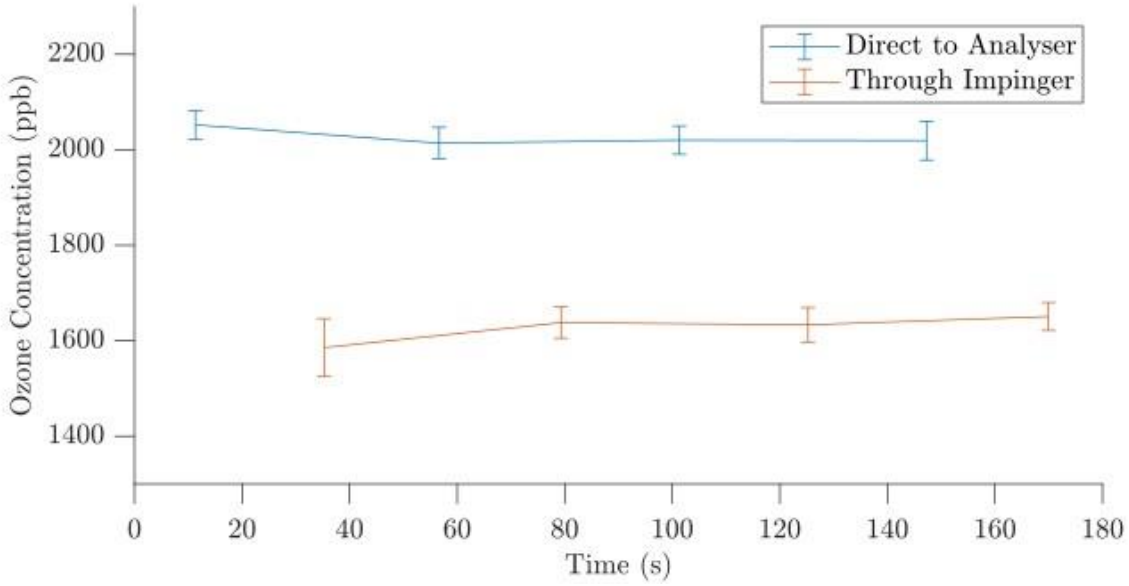

Supplementary Figure 3. Plot demonstrating the collection efficiency of ozone in solution. The three way valve was switched every 20 minutes to allow the signal to stabilize. The collection efficiency was calculated to be 21.0 % ± 3.9.

BPEA in DMSO Reaction with Ozone LC-MS chromatograms

HPLC plots from hourly samples of 0.5 mM BPEA in DMSO being bubbled with ~9.5 ppm of ozone over a 6-hour period. The resulting chromatograms monitored the fluorescence emission at 492 nm, UV absorption at 434 nm and 254 nm.

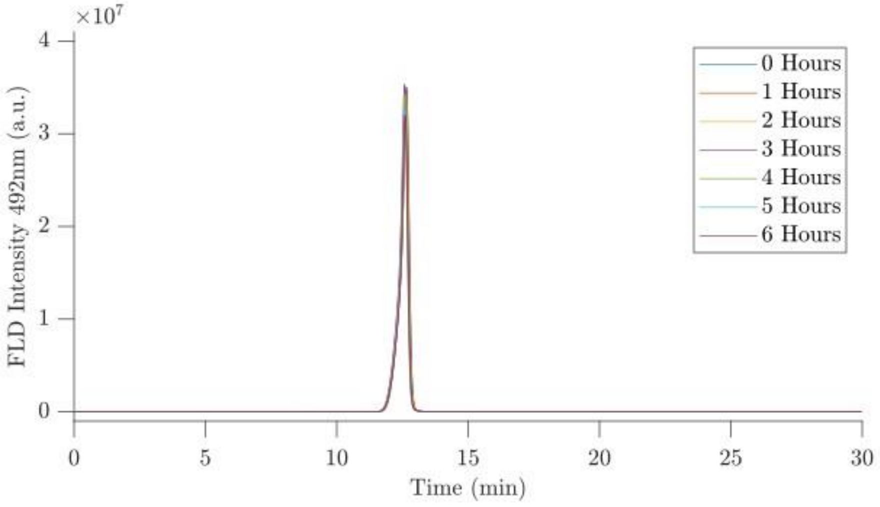

Supplementary Figure 4. Fluorescence chromatogram from hourly sampling of BPEA/DMSO bubbled with ~9.5 ppm of ozone.

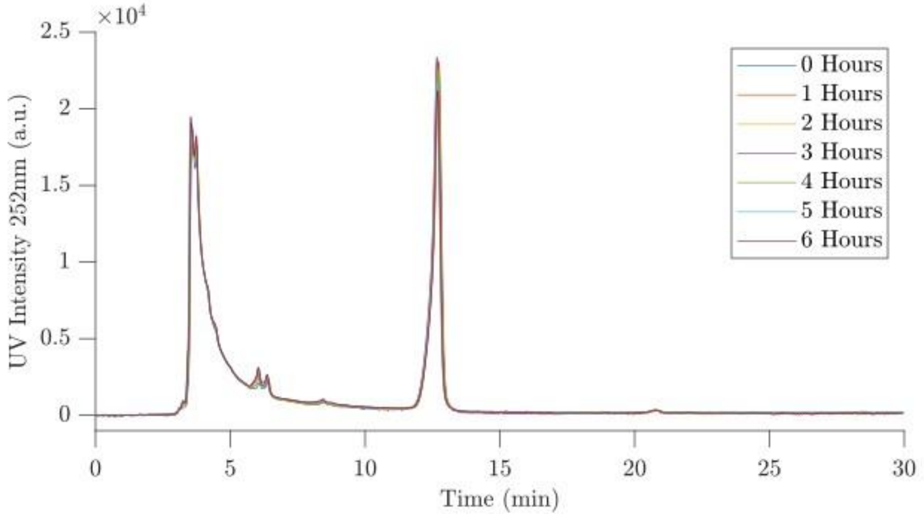

Supplementary Figure 5. UV chromatogram at 252 nm from hourly sampling of BPEA/DMSO bubbled with ~ 9.5 ppm of ozone. The trailing peak at ~4 min corresponds to DMSO.

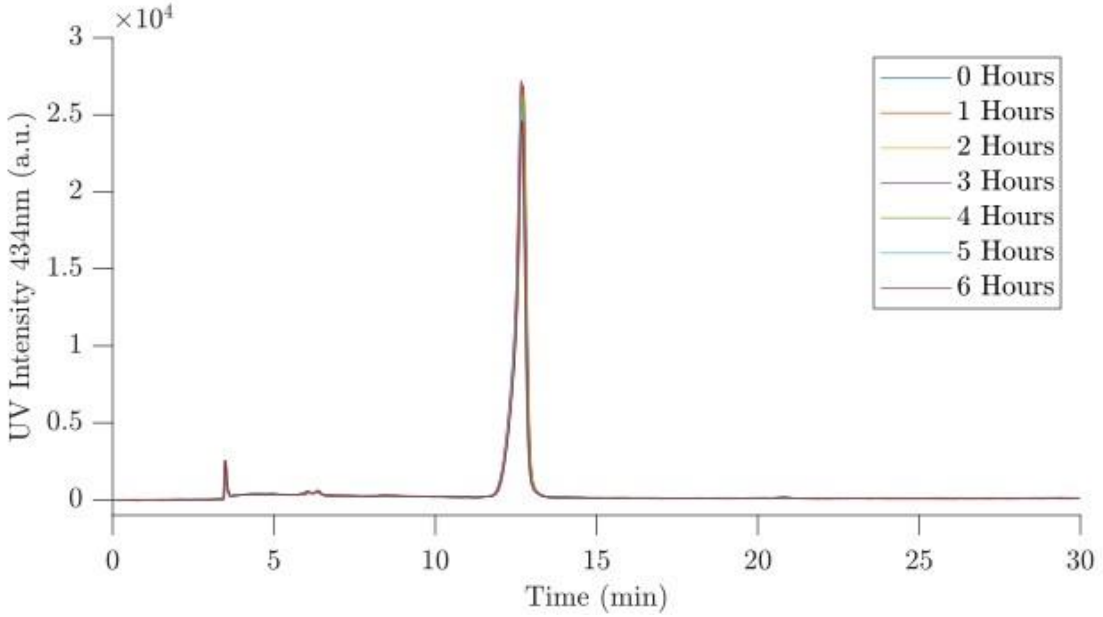

Supplementary Figure 6. UV chromatogram at 434nm from hourly sampling of BPEA/DMSO bubbled with ~9.5 ppm ozone.

BPEAnit in DMSO Reaction with Ozone Hourly Sample Chromatograms

LC-MS results of BPEAnit (0.5 mM) in DMSO bubbled with 9.5 ppm of ozone with hourly samples being taken from the reaction mixture.

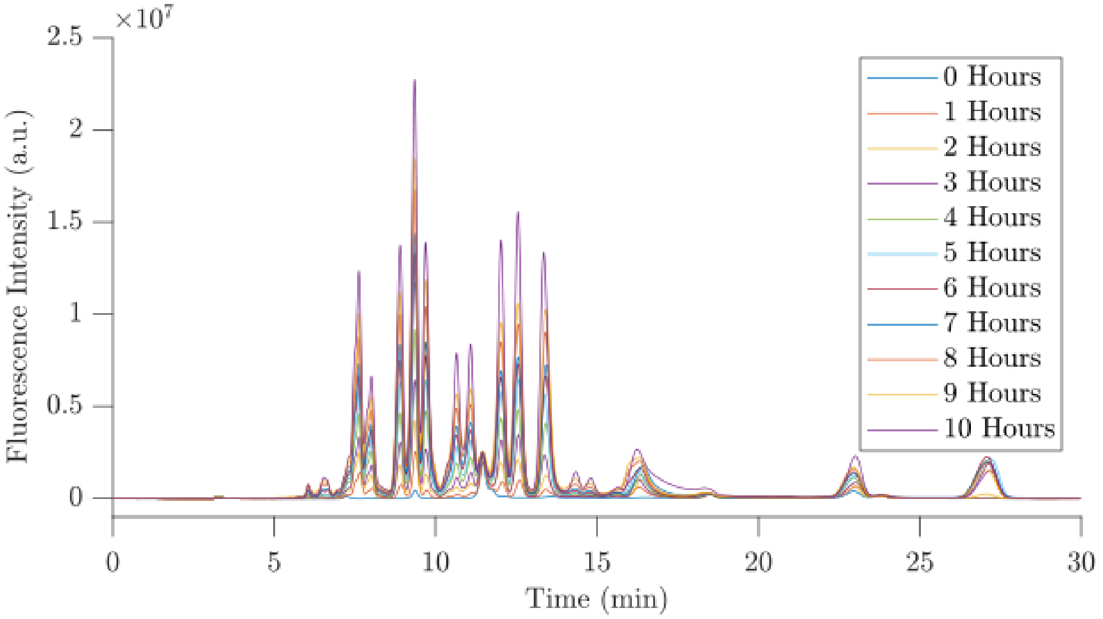

Supplementary Figure 7: Fluorescence HPLC trace (λ_{ex} = 434 nm, λ_{em} = 492 nm) during the 10-hour ozone bubbling period of BPEAnit in DMSO with hourly sampling.

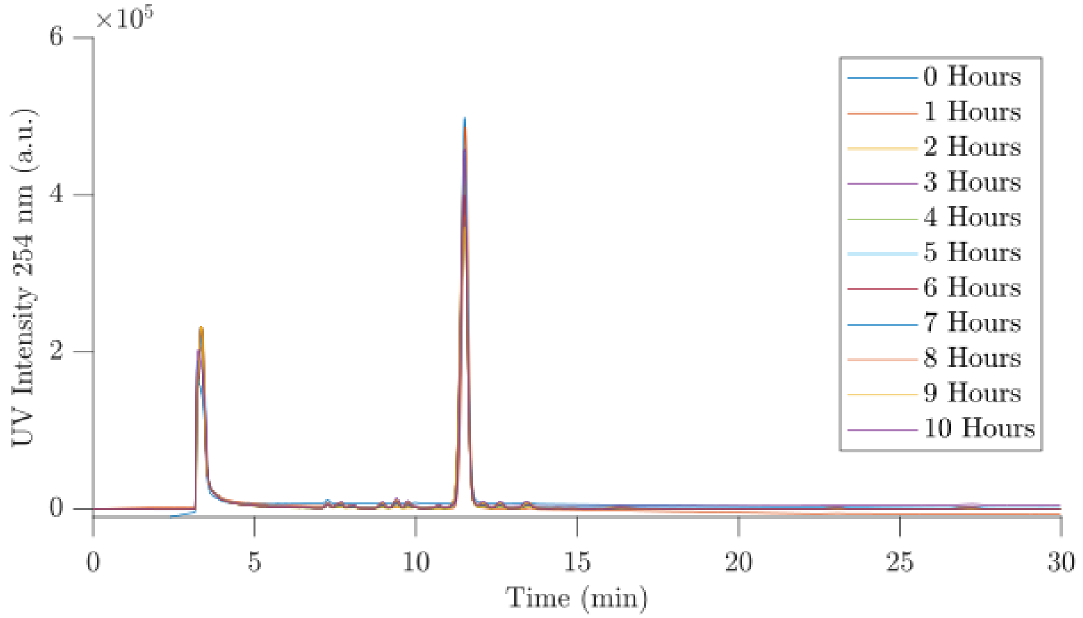

Supplementary Figure 8: UV HPLC trace (abs. 254 nm) during the 10-hour ozone bubbling period of BPEAnit in DMSO with hourly sampling.

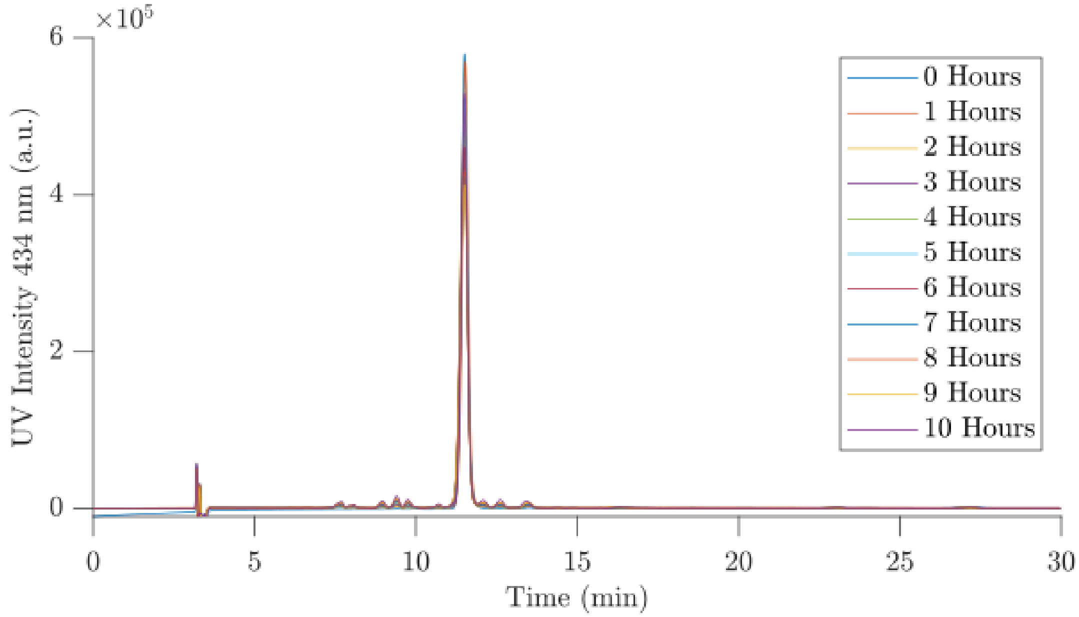

Supplementary Figure 9: UV HPLC trace (abs. 434 nm) during the 10-hour ozone bubbling period of BPEAnit in DMSO with hourly sampling.

DMSO Reaction with Ozone Hourly Sample Chromatograms

DMSO was bubbled with 9.5ppm of ozone for 6 hours as per the reported method. These figures are to illustrate that there is no product formation measured when ozone and DMSO are combined without BPEAnit present. Samples were ran using the LC-MS general method outlined in the methods section.

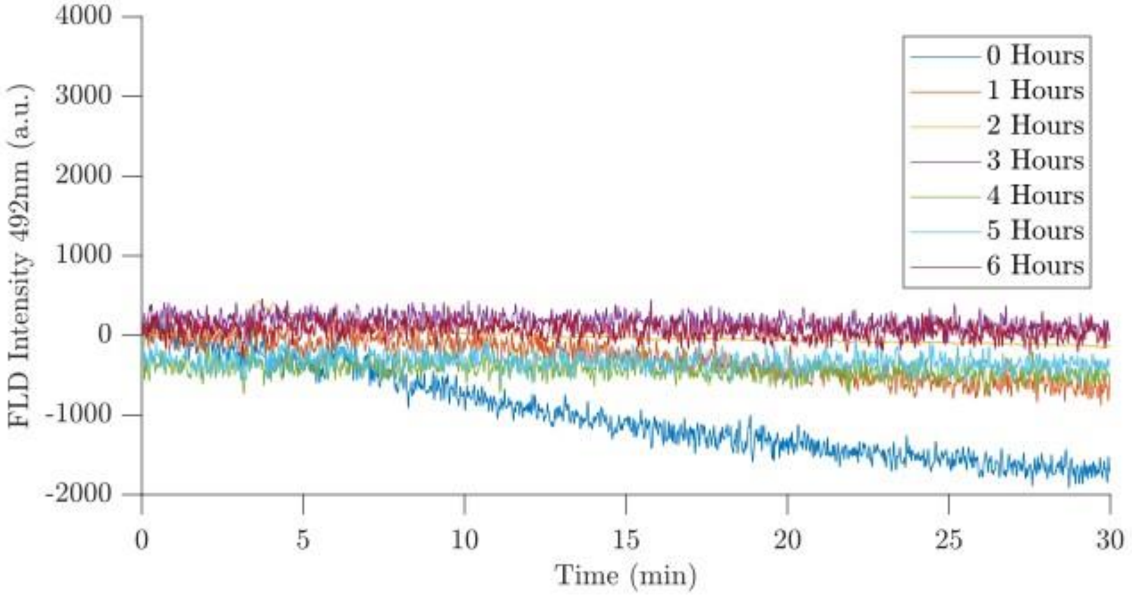

Supplementary Figure 10: Fluorescence HPLC trace (λ_{ex} = 434 nm, λ_{em} = 492 nm) of DMSO bubbled with ozone for 6 hours with hourly sampling.

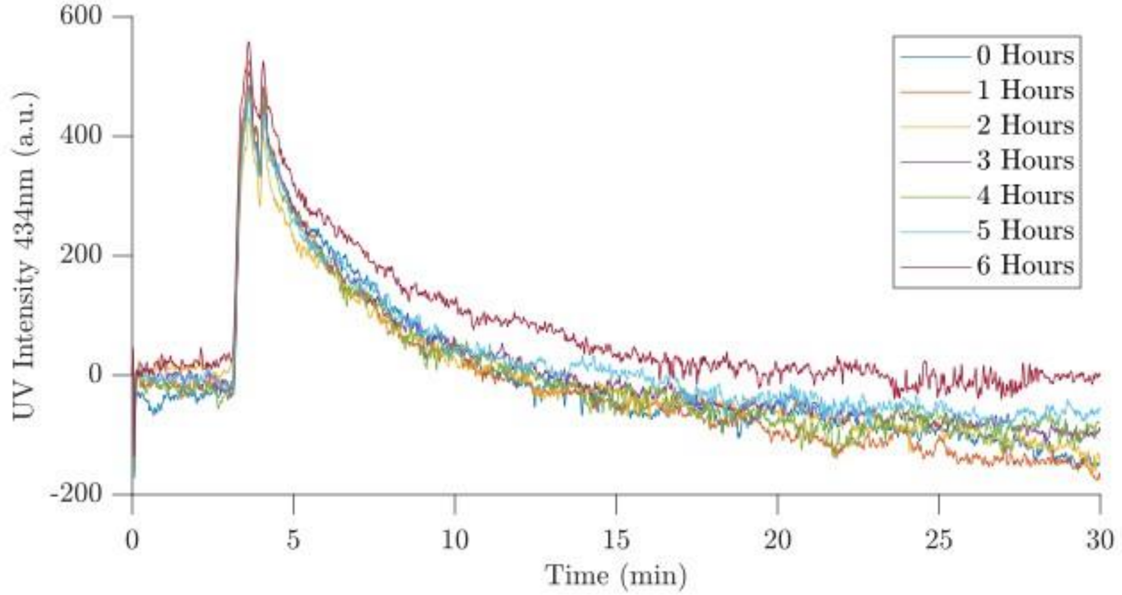

Supplementary Figure 11: UV HPLC trace (abs. 434 nm) of DMSO bubbled with ozone for 6 hours with hourly sampling.

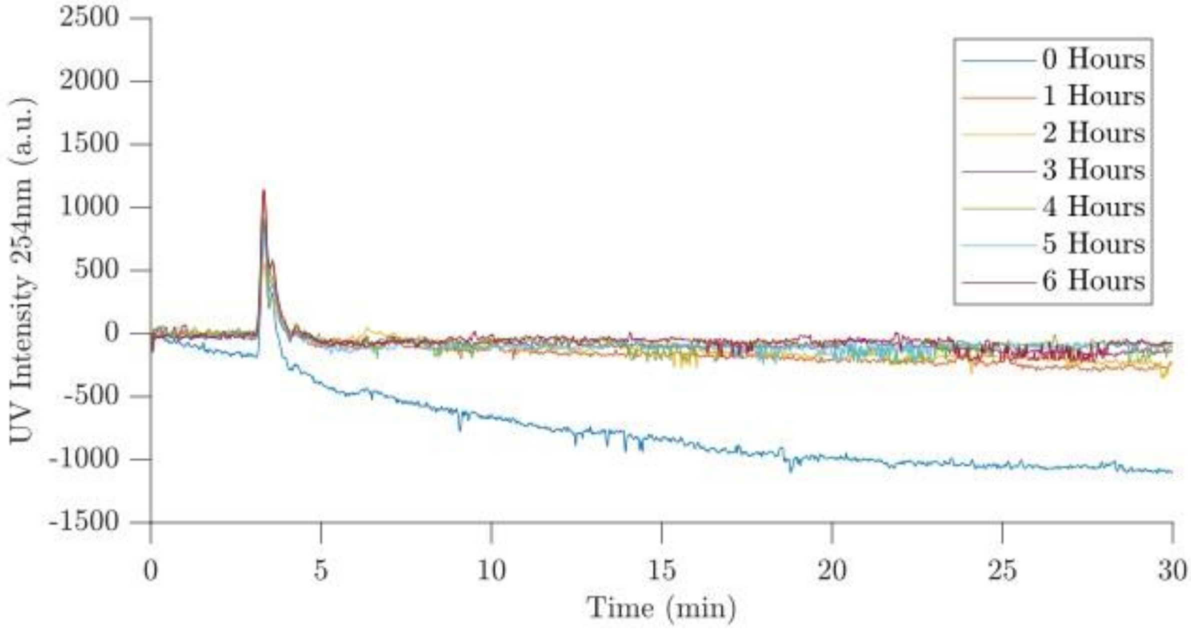

Supplementary Figure 12: UV HPLC trace (abs. 254 nm) of DMSO bubbled with ozone for 6 hours with hourly sampling.

Molecular Ion Identification and Mass Spectra for BPEAnit-derived Products

To confirm the reproducibility of the data, the ozone sampling experiment with BPEAnit was conducted in triplicate and subsequently analysed by LC-MS. The following plots demonstrate the fluorescence chromatogram of replicate 3, as well as the corresponding intensity for the identified *m*/*z*, followed by the mass spectrum for the specified peak across each of the replicates. The MS intensity plots were smoothed using Gaussian smoothing. The minor peak visible at ~27 min was identified as a small (<1%) impurity remaining from the synthesis of BPEAnit and as such was not included in our product analysis.

Peak A

m/*z* = 539.2084, r.t. = 7.62 min

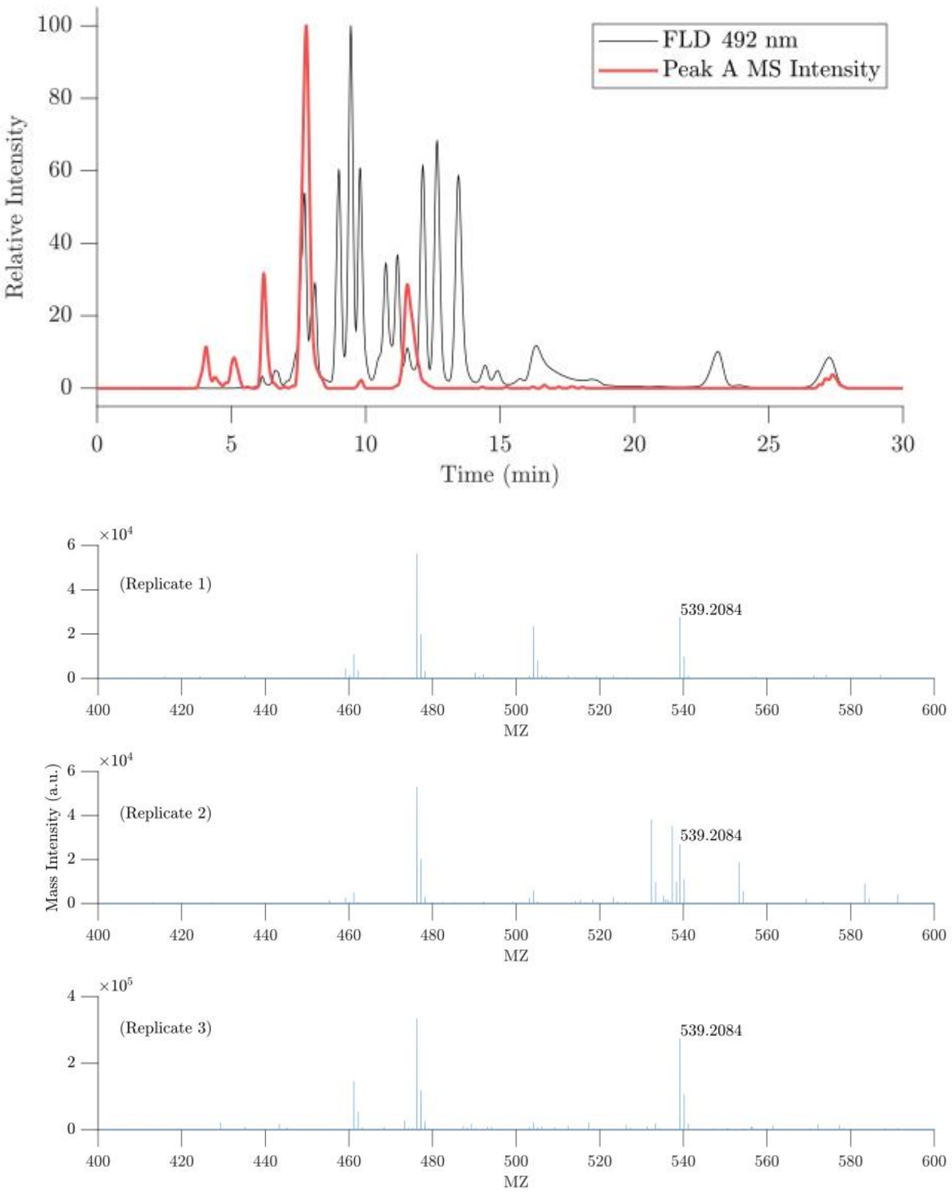

Supplementary Figure 13: Fluorescence HPLC-MS trace (λ_{ex} = 434 nm, λ_{em} = 492 nm) of 0.5 mM BPEAnit in DMSO after 10 h exposure to a constant stream of ozone (ca. 9.5 ppm) overlayed with the mass intensity plot for the identified *m*/*z* 539.2084 ± 5 ppm (top). Obersved *m*/*z* across replicates (bottom).

Peak B / Compound 1

m/z = 504.1958, r.t. = 8.01 min

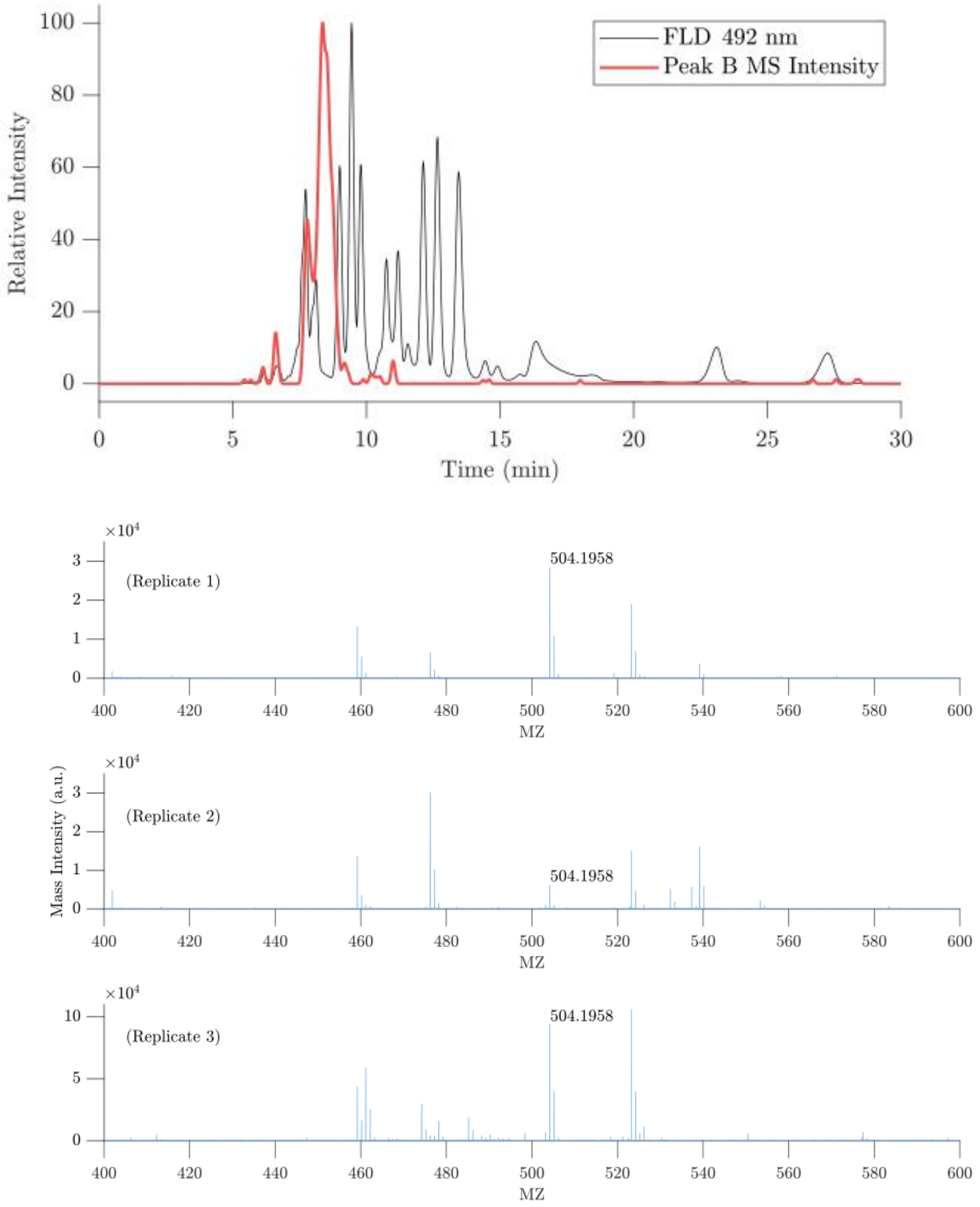

Supplementary Figure 14: Fluorescence HPLC-MS trace (λ_{ex} = 434 nm, λ_{em} = 492 nm) of 0.5 mM BPEAnit in DMSO after 10 h exposure to a constant stream of ozone (ca. 9.5 ppm) overlayed with the mass intensity plot for the identified *m/z* 504.1958 ± 5 ppm (top). Obersved *m/z* across replicates (bottom).

Peak C / Compound 2

m/z = 569.2019, r.t. = 8.90 min

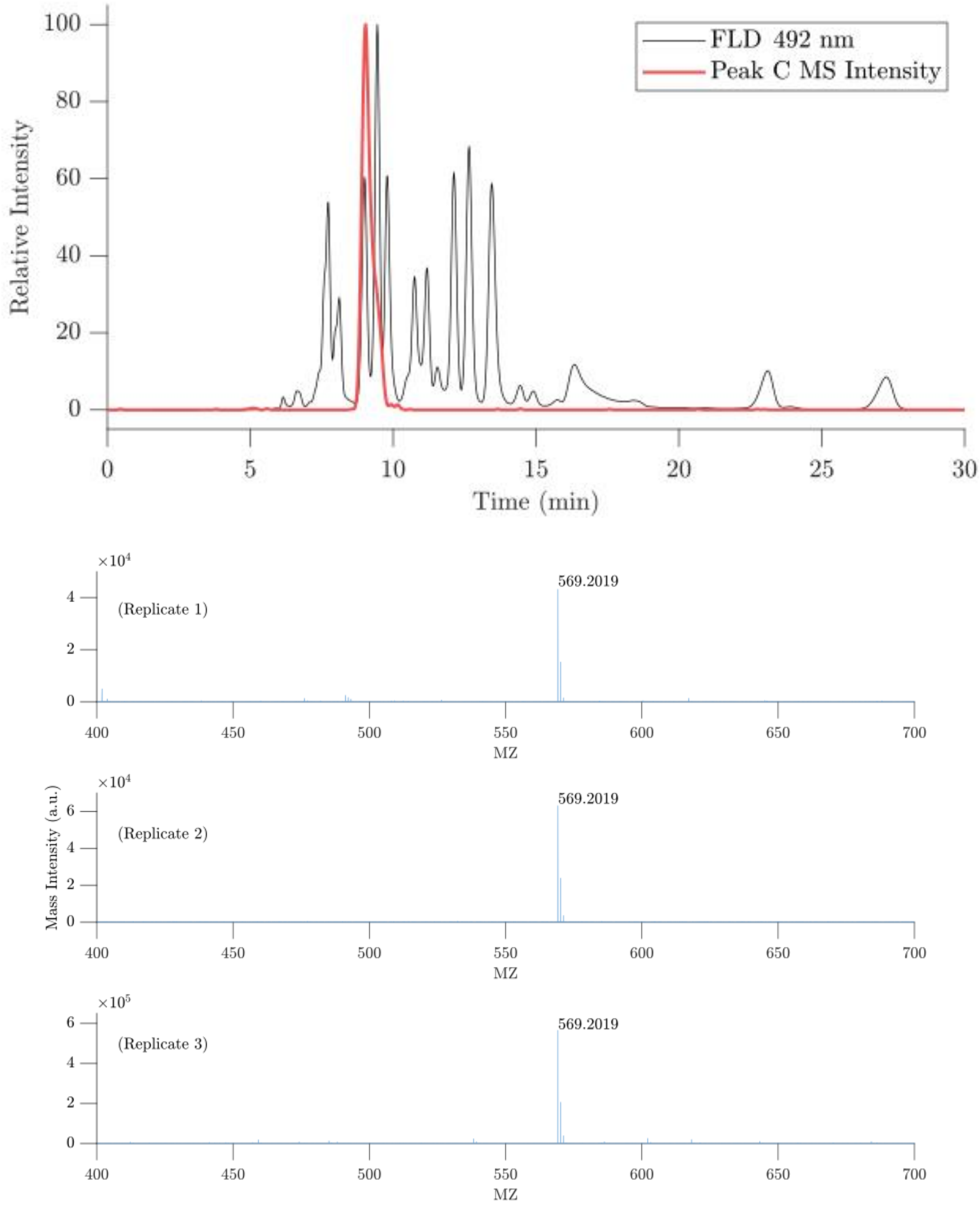

Supplementary Figure 15: Fluorescence HPLC-MS trace (λ_{ex} = 434 nm, λ_{em} = 492 nm) of 0.5 mM BPEAnit in DMSO after 10 h exposure to a constant stream of ozone (ca. 9.5 ppm) overlayed with the mass intensity plot for the identified *m/z* 569.2019 ± 5 ppm (top). Obersved *m/z* across replicates (bottom).

Peak D / Compound 3

m/z = 553.2070, r.t. = 9.35 min

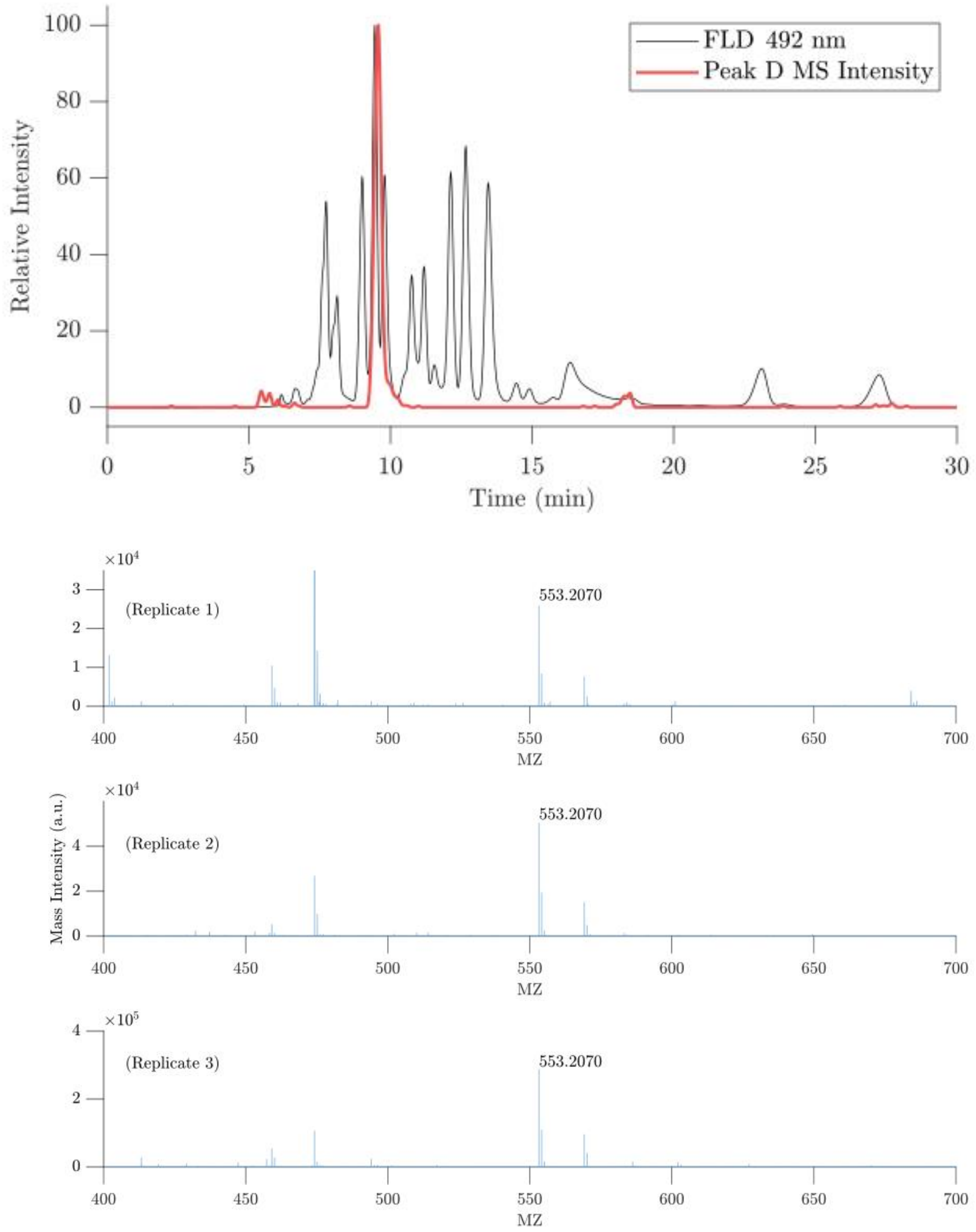

Supplementary Figure 16: Fluorescence HPLC-MS trace (λ_{ex} = 434 nm, λ_{em} = 492 nm) of 0.5 mM BPEAnit in DMSO after 10 h exposure to a constant stream of ozone (ca. 9.5 ppm) overlayed with the mass intensity plot for the identified *m/z* 553.2070 ± 5 ppm (top). Obersved *m/z* across replicates (bottom).

Peak E / Compound 4

m/z = 476.2006, r.t. = 9.69 min

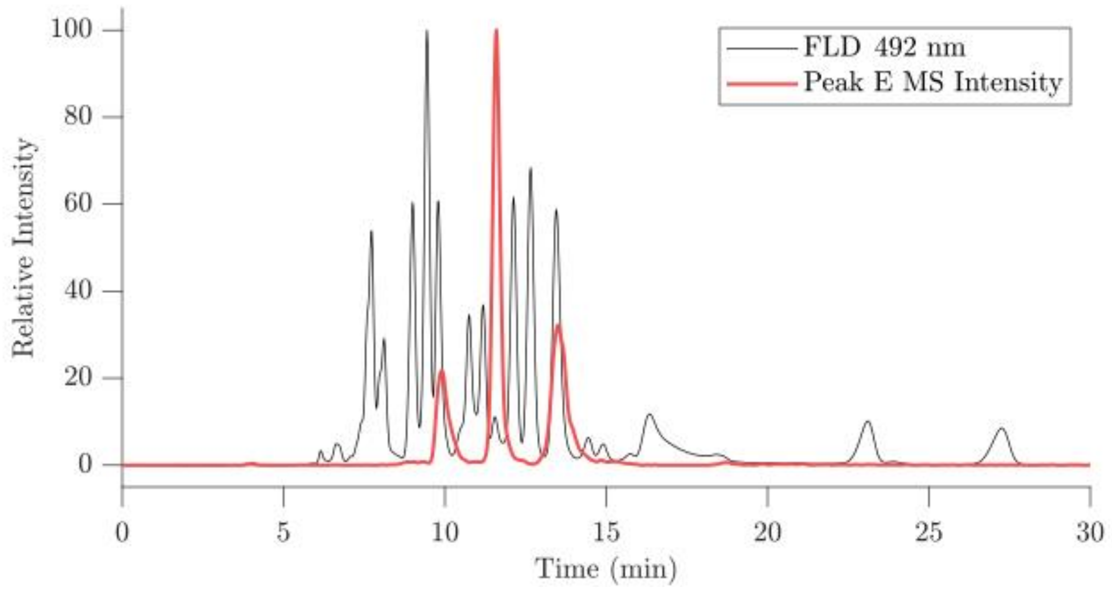

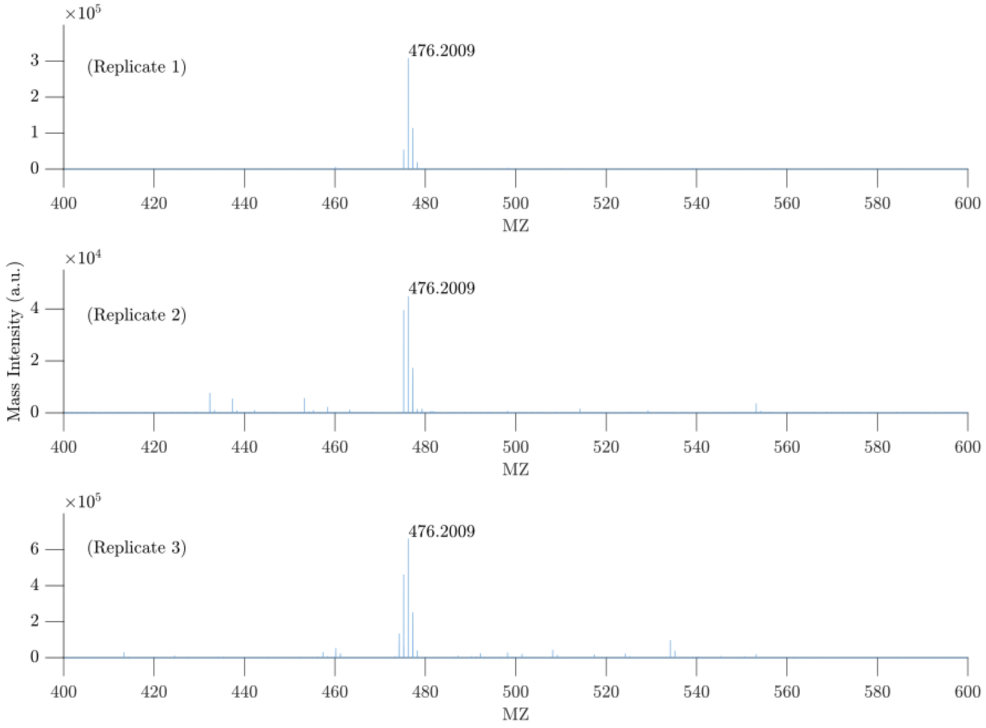

Supplementary Figure 17: Fluorescence HPLC-MS trace (λ_{ex} = 434 nm, λ_{em} = 492 nm) of 0.5 mM BPEAnit in DMSO after 10 h exposure to a constant stream of ozone (ca. 9.5 ppm) overlayed with the mass intensity plot for the identified *m/z* 476.2006 ± 5 ppm (top). Obersved *m/z* across replicates (bottom).

Peak F

m/z = 505.2039, r.t. = 10.65 min

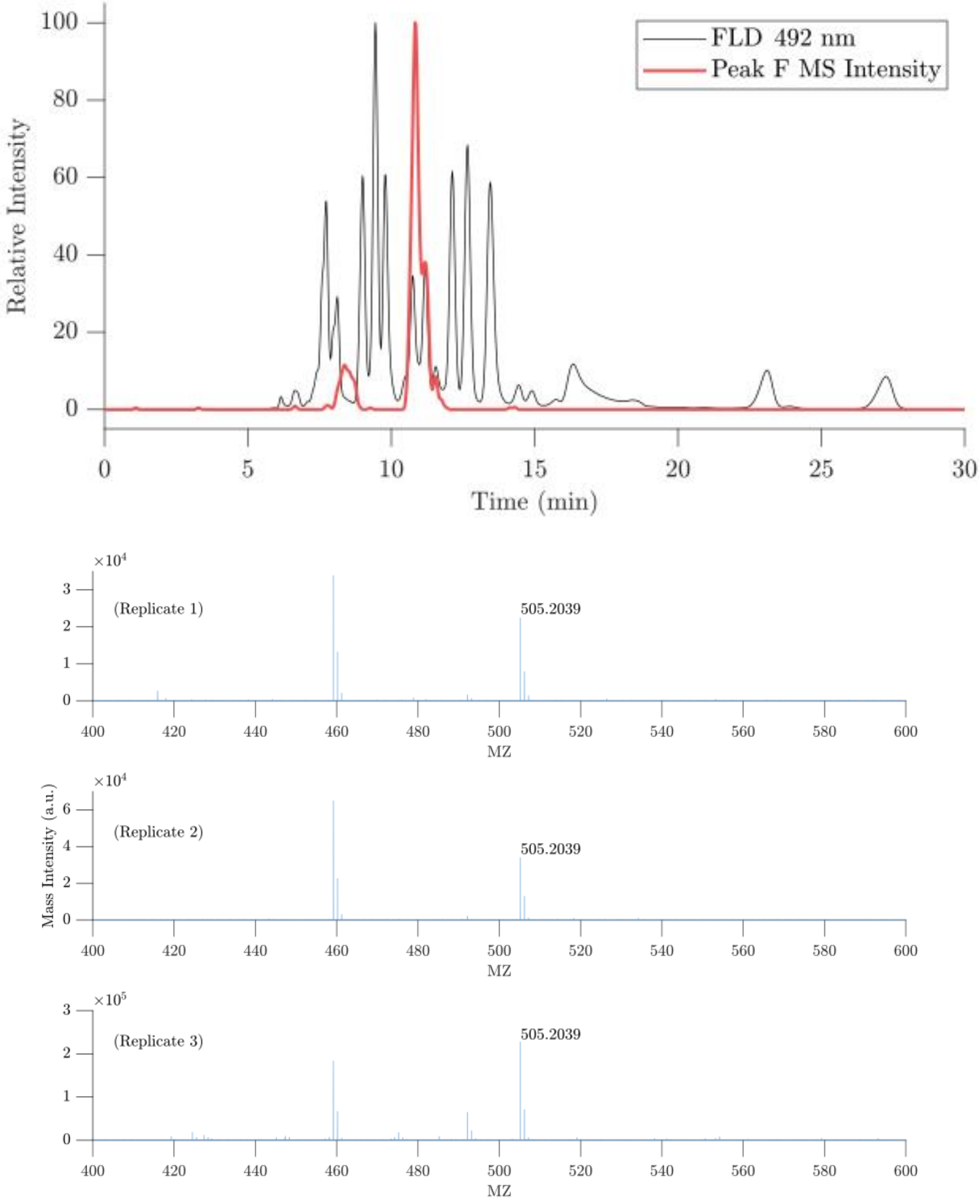

Supplementary Figure 18: Fluorescence HPLC-MS trace (λ_{ex} = 434 nm, λ_{em} = 492 nm) of 0.5 mM BPEAnit in DMSO after 10 h exposure to a constant stream of ozone (ca. 9.5 ppm) overlayed with the mass intensity plot for the identified *m/z* 505.2039 ± 5 ppm (top). Obersved *m/z* across replicates (bottom).

Peak H / BPEAnit

m/*z* = 490.2165, r.t. = 11.45 min

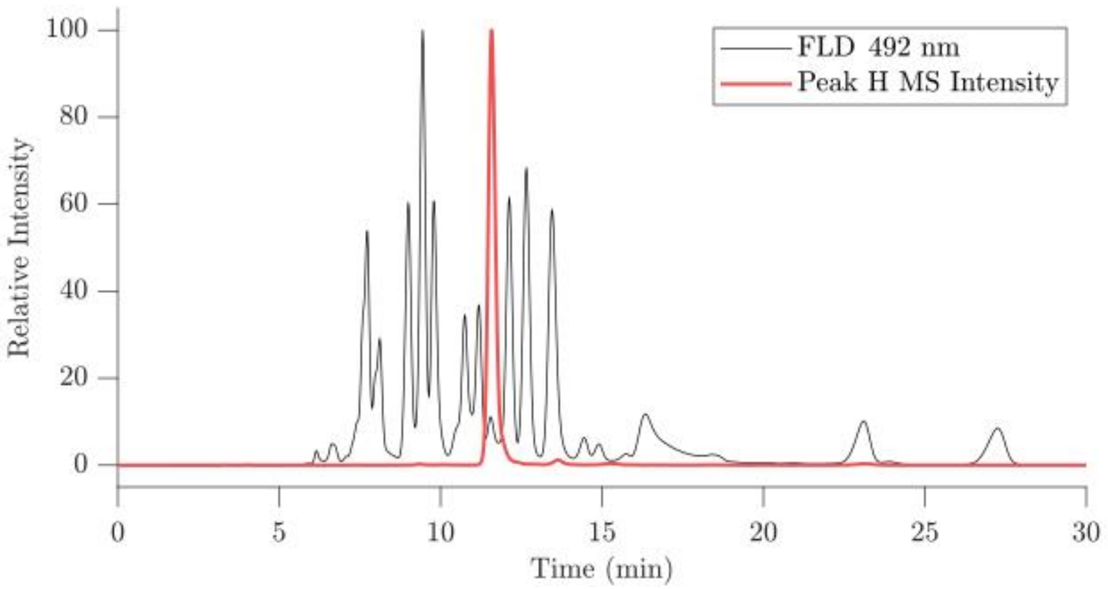

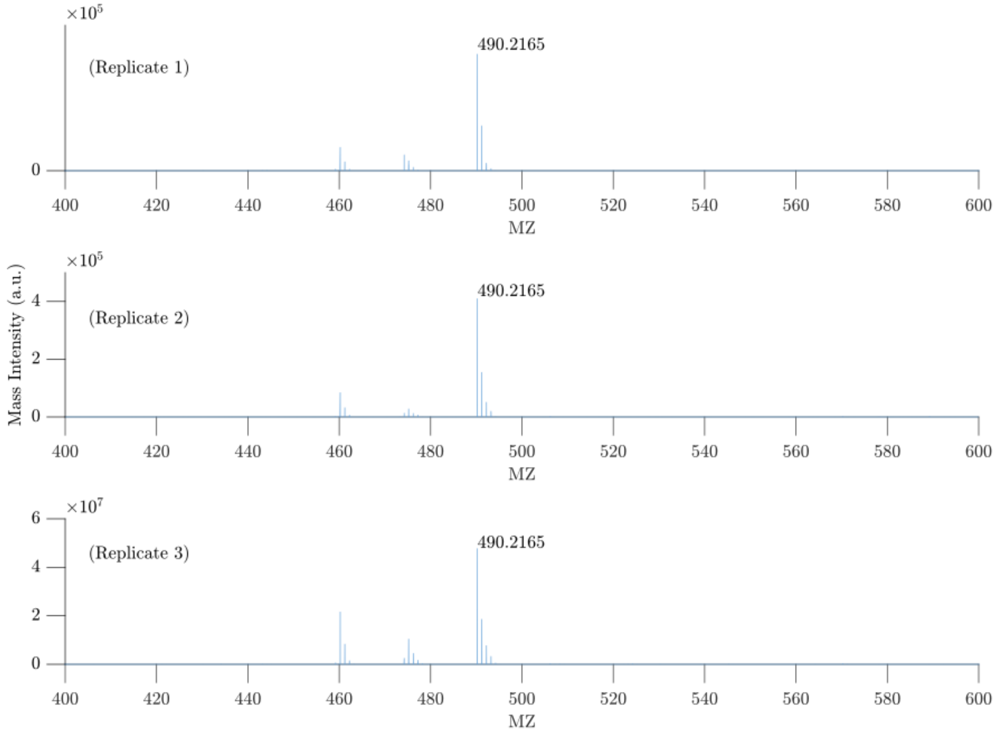

Supplementary Figure 19: Fluorescence HPLC-MS trace (λ_{ex} = 434 nm, λ_{em} = 492 nm) of 0.5 mM BPEAnit in DMSO after 10 h exposure to a constant stream of ozone (ca. 9.5 ppm) overlayed with the mass intensity plot for the identified *m/z* 490.2165 ± 5 ppm (top). Obersved *m/z* across replicates (bottom).

Peak K

m/z = 504.2322, r.t. = 13.35 min

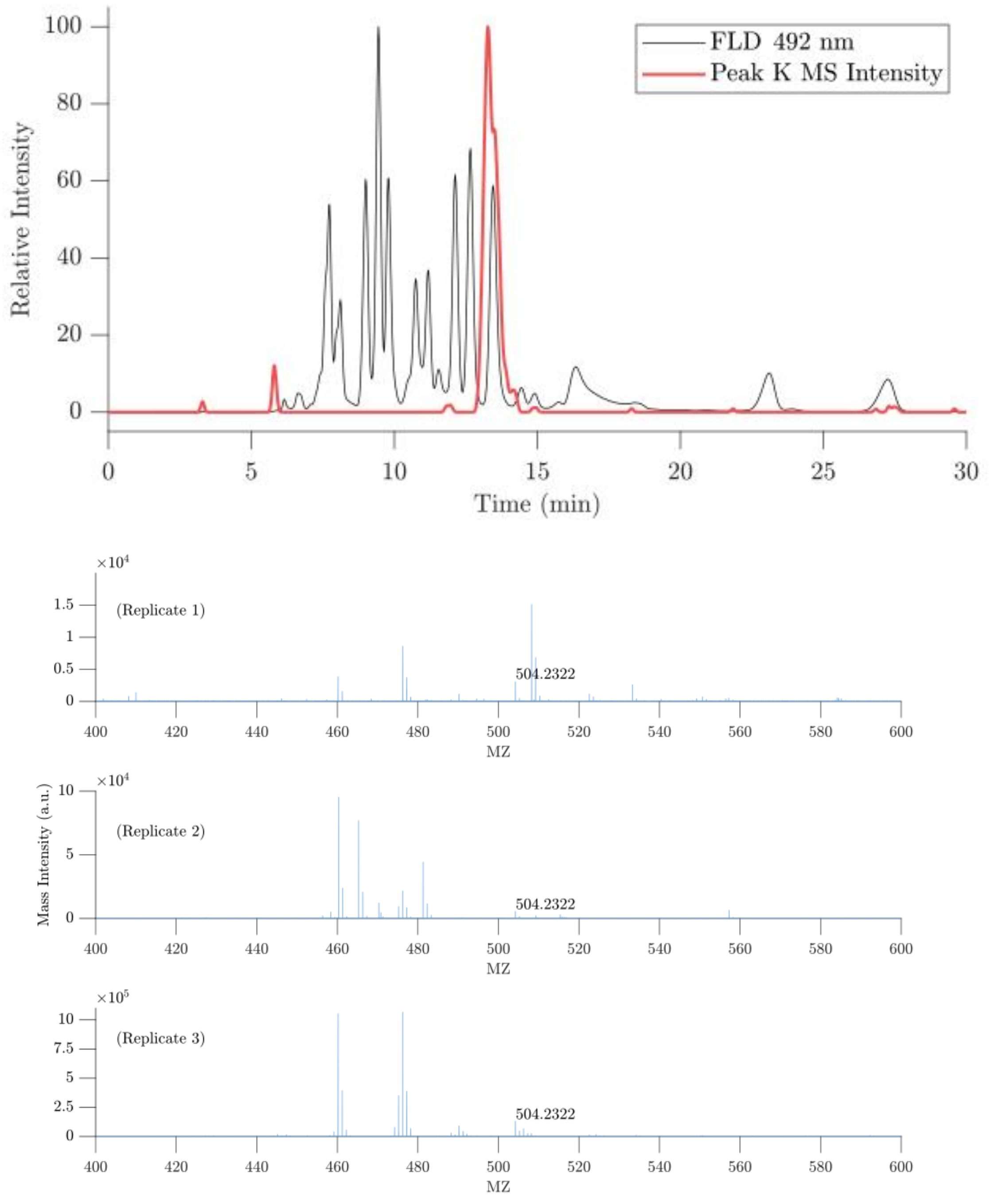

Supplementary Figure 20: Fluorescence HPLC-MS trace (λ_{ex} = 434 nm, λ_{em} = 492 nm) of 0.5 mM BPEAnit in DMSO after 10 h exposure to a constant stream of ozone (ca. 9.5 ppm) overlayed with the mass intensity plot for the identified *m/z* 504.2322 ± 5 ppm (top). Obersved *m/z* across replicates (bottom).

Peak L / Compound 5

m/*z* = 568.2305, r.t. = 16.24 min

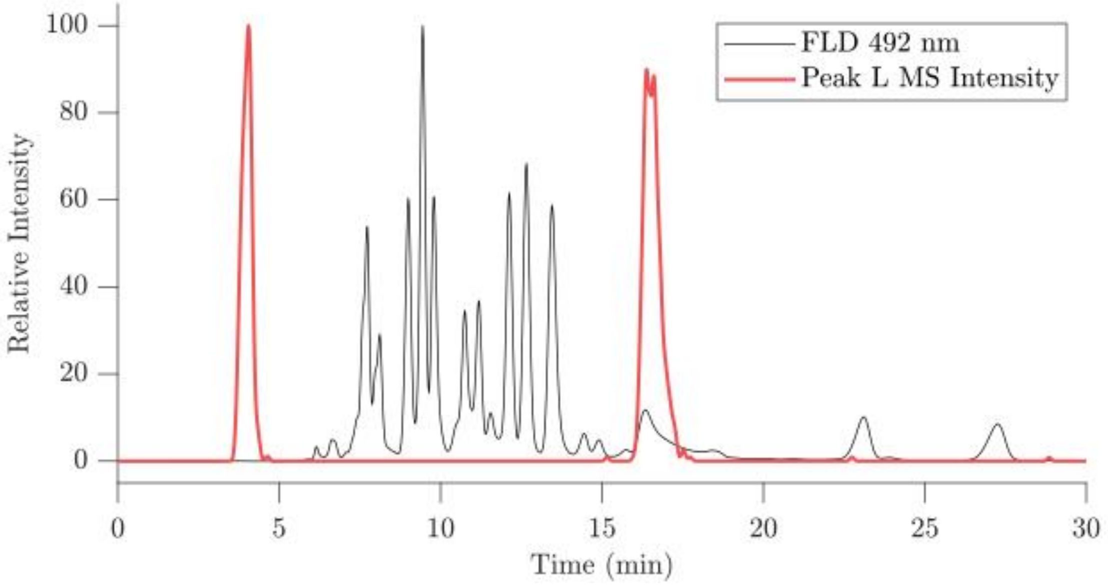

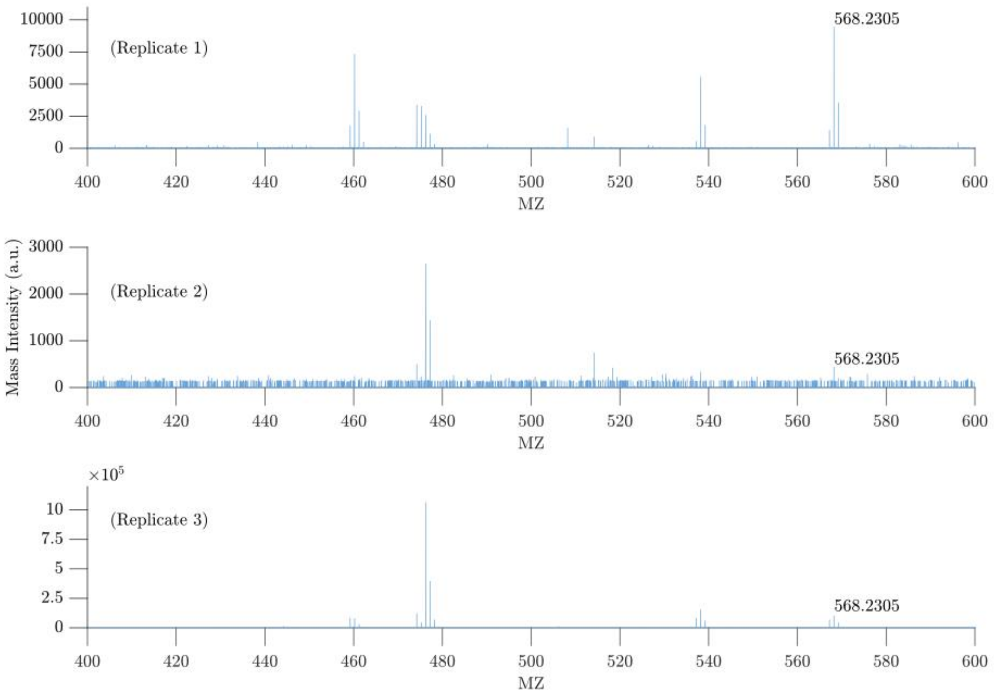

Supplementary Figure 21: Fluorescence HPLC-MS trace (λ_{ex} = 434 nm, λ_{em} = 492 nm) of 0.5 mM BPEAnit in DMSO after 10 h exposure to a constant stream of ozone (ca. 9.5 ppm) overlayed with the mass intensity plot for the identified *m/z* 568.2305 ± 5 ppm (top). Obersved *m/z* across replicates (bottom).

Peak M, Compound 6

m/*z* = 506.2476, r.t. = 23.00 min

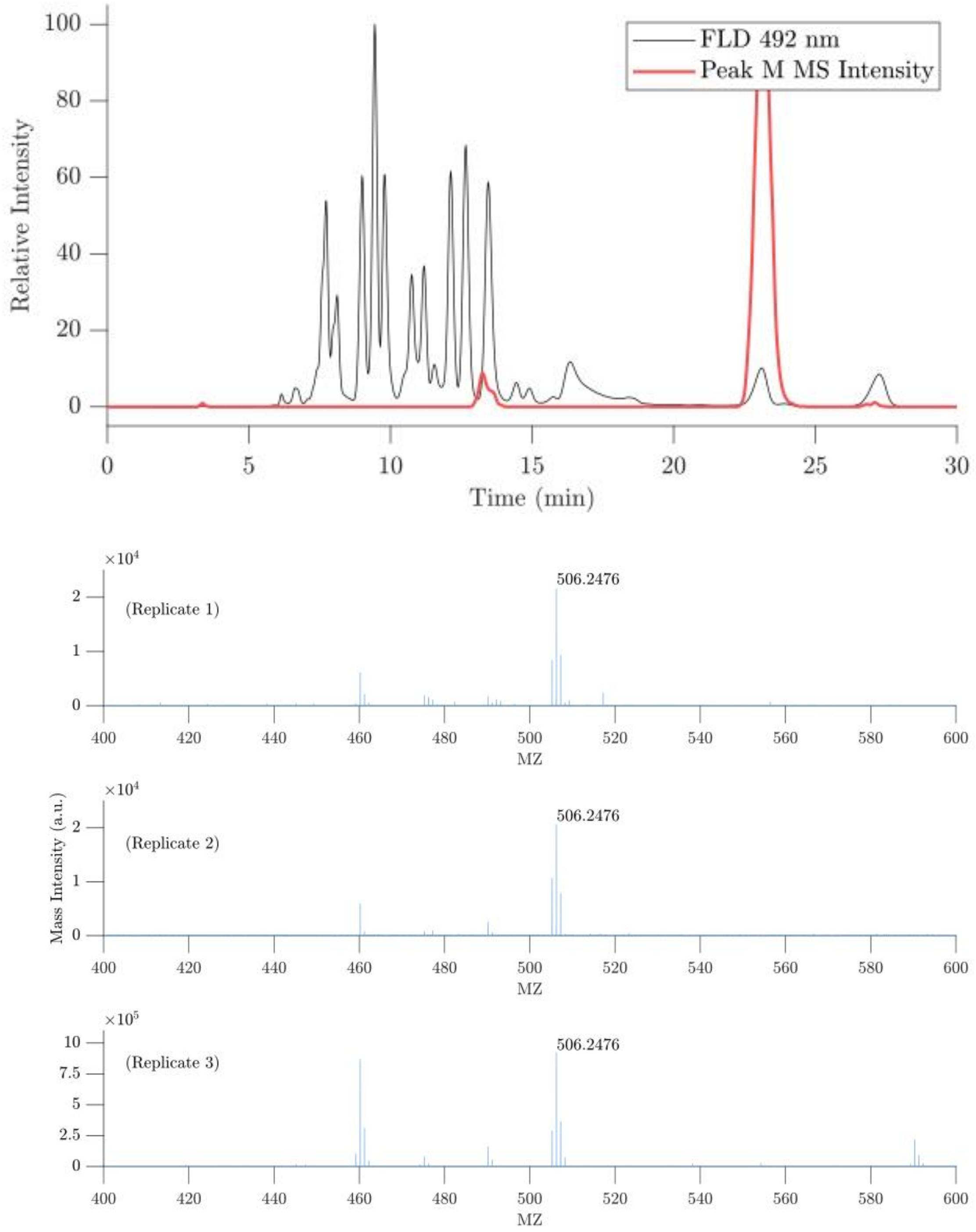

Supplementary Figure 22: Fluorescence HPLC-MS trace (λ_{ex} = 434 nm, λ_{em} = 492 nm) of 0.5 mM BPEAnit in DMSO after 10 h exposure to a constant stream of ozone (ca. 9.5 ppm) overlayed with the mass intensity plot for the identified *m/z* 506.2476 ± 5 ppm (top). Obersved *m/z* across replicates (bottom).

TMIO (Parent Nitroxide) Reaction with Ozone

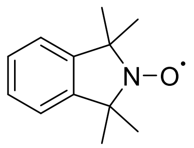

TMIO

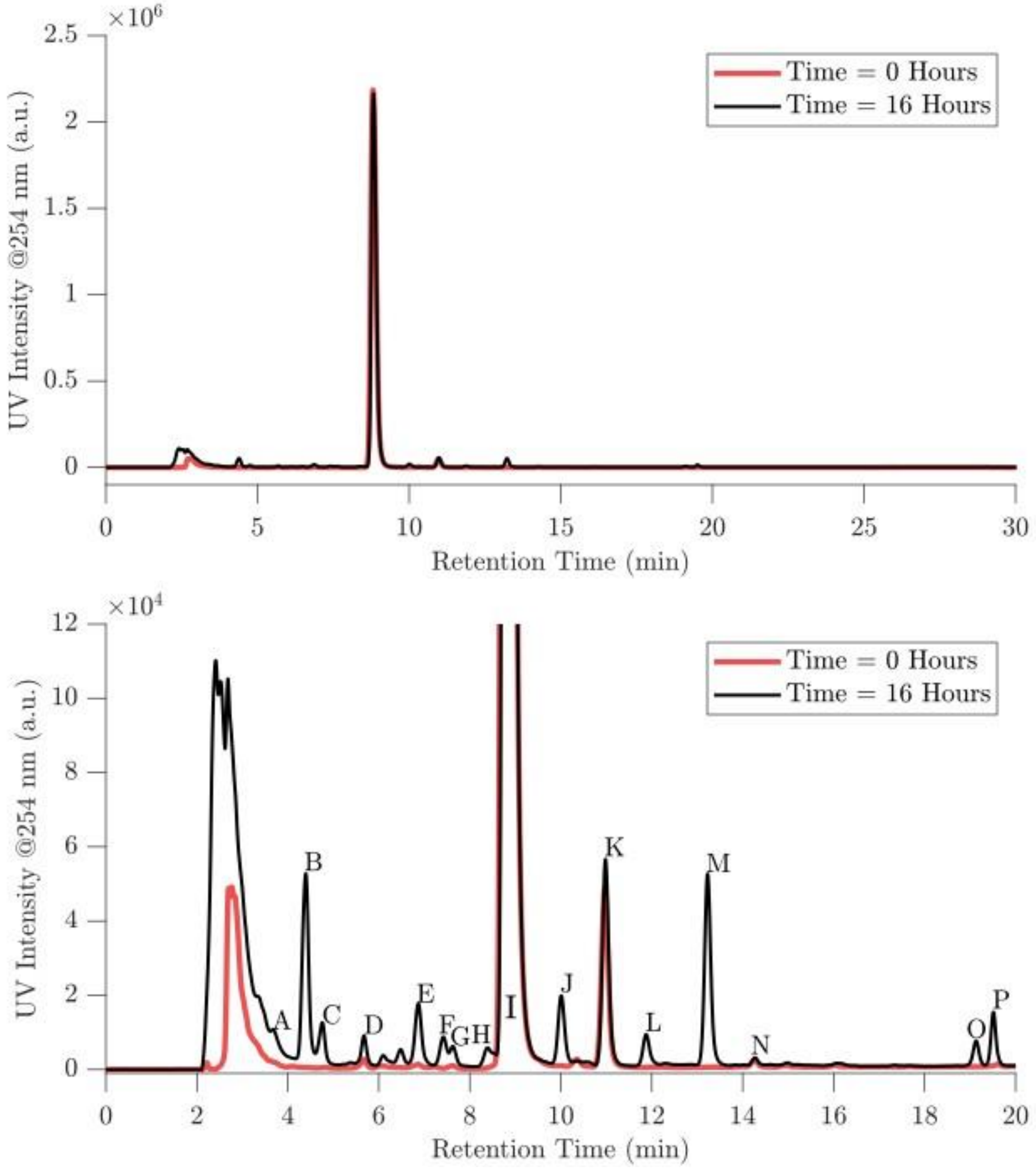

Supplementary Figure 23: UV HPLC-MS trace (abs. 254 nm) of TMIO (35 mM) in DMSO bubbled with 9.5 ppm ozone for 16 hours with hourly sampling.

Supplementary Table 1: LC-MS results for the reaction of TMIO (35 mM) in DMSO bubbled with 9.5 ppm ozone for 16 hours.

Peak	Retention Time (min)	Observed m/z	Closest Assigned Molecular Formula	Δm/z (ppm)	Assigned Structure
A	3.67	204.1018	$C_{12}H_{14}NO_2$ 204.1019 $[M+H]^+$	-0.49	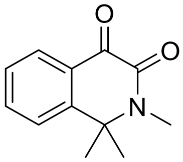
B	4.39	176.1071	$C_{11}H_{14}NO$ 176.1070 $[M+H]^+$	0.57	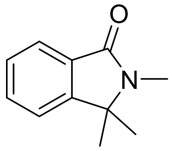
C	4.76	241.0949	$C_{11}H_{15}NO_5$ 241.095	-0.41	-
D	5.67	238.1244	$C_{13}H_{20}NOS$ 238.1260	-6.72	-
E	6.87	254.1192	$C_{13}H_{20}NO_2S$ 254.1209 $[M+H]^+$	-6.69	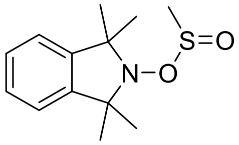
F	7.42	278.0771	-	-	-
G	7.63	-	-	-	-
H	8.39	192.1368	$C_{12}H_{18}NO$ 192.1383	-7.81	-
**I**	8.82	190.1215	$C_{12}H_{16}NO$ 190.1226 $[M]$	-5.79	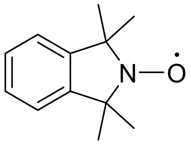
J	10.01	-	-	-	-
K	10.98	290.1731	290.1751	-6.89	-
L	11.88	270.1143	$C_{13}H_{20}NO_3S$ 270.1158 $[M+H]^+$	-5.55	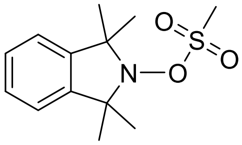
M	13.23	215.1527	-	-	-
N	14.27	204.1372	$C_{13}H_{18}NO$ 204.1383	-5.39	-
O	19.13	206.1525	$C_{13}H_{20}NO$ 206.1539 $[M+H]^+$	-6.79	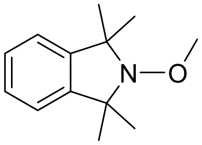
P	19.51	413.2411	$C_{24}H_{33}N_2O_4$ 413.2435	-5.81	-

BPEAnit in DMSO Reaction with Ozone (Increased Water Content)

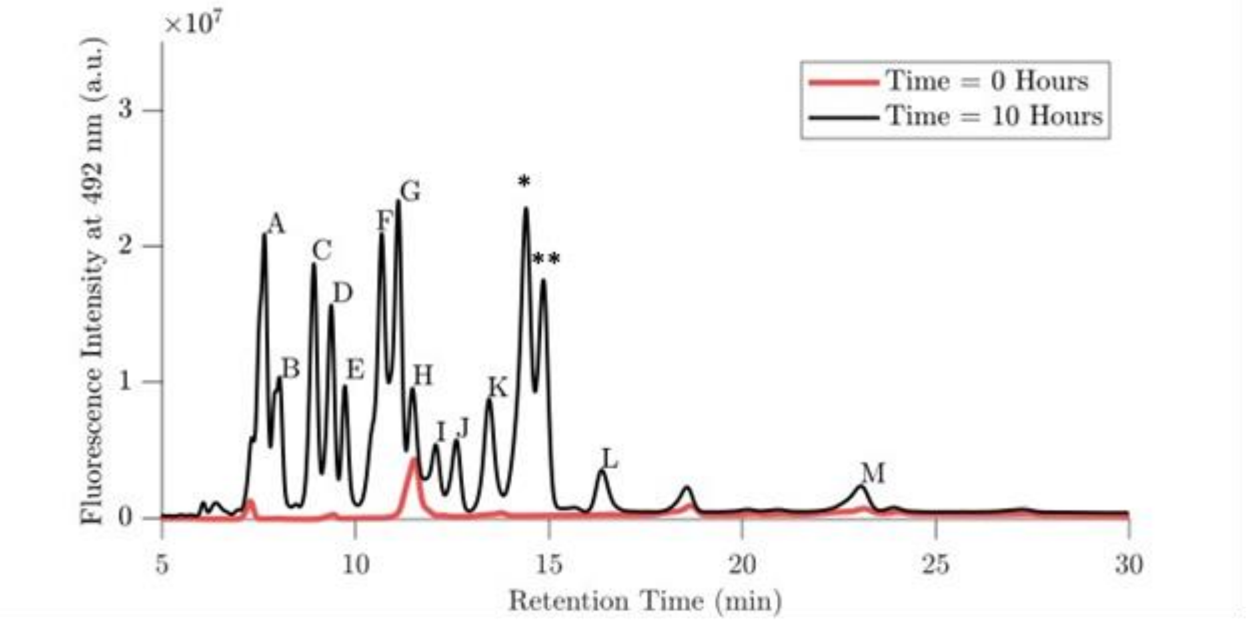

Supplementary Figure 24: Fluorescence HPLC-MS trace (λ_{ex} = 434 nm, λ_{em} = 492 nm) of 0.5 mM BPEAnit in DMSO spiked with water after 10 h exposure to a constant stream of ozone (ca. 9.5 ppm). Water content was determined to be 4279 ppm by coulometric titration.

Supplementary Table 2: LC-MS results for the reaction of 0.5 mM BPEAnit in DMSO spiked with water after 10 h exposure to a constant stream of ozone (ca. 9.5 ppm).

Peak	Retention Time (min)	Observed m/z	Closest Assigned Molecular Formula	Δm/z (ppm)
A	7.6	539.2089	$C_{36}H_{29}NO_4$ (539.2086)	0.56
B	8.0	504.1956	$C_{36}H_{26}NO_2$ (504.1958)	0.40
C	8.9	569.2017	$C_{37}H_{31}NO_3S$ (569.2019)	0.35
D	9.3	553.2069	$C_{37}H_{31}NO_2S$ (553.2070)	0.18
E	9.7	476.2005	$C_{35}H_{26}NO$ (476.2009)	0.84
F	10.6	505.2034	$C_{36}H_{27}NO_2$ (505.2042)	1.58
G	11.1	-[a]	-	-
**H**	11.4	490.2164	**$C_{36}H_{28}NO$ (490.2165)**	0.20
I	12.0	-[a]	-	-
J	12.6	-[a]	-	-
K	13.4	504.2321	$C_{37}H_{30}NO$ (504.2322)	0.19
*	14.3	602.1099	-[b]	-
**	14.9	602.1099	-[b]	-
L	16.3	568.2302	$C_{38}H_{34}NO_2S$ (568.2305)	0.52
M	23.0	506.2473	$C_{37}H_{32}NO$ (506.2478)	0.99
N	27.2	520.2631	$C_{38}H_{34}NO$ (520.2635)	0.77

[a] No predominant ion observed. [b] No sensible molecular formula could be deduced from the observed *m/z*.

Supplementary Table 3: Change in FLD% peak area relative to H_2O concentration in DMSO after 10 h exposure to ozone. Changes of most significance are highlighted. Peaks were analysed for area% in Xcalibur 3.0.

Peak	DMSO		Peak	DMSO	
	396 ppm H_2O (FLD%)	4279 ppm H_2O (FLD%)		396 ppm H_2O (FLD%)	4279 ppm H_2O (FLD%)
**A**	9.4	10.8	I	10.1	2.8
**B**	4.6	4.8	J	11.0	3.1
**C**	10.3	7.4	K	8.9	5.3
**D**	17.4	6.1	L	1.2	2.3
**E**	9.9	3.5	M	0.1	1.8
**F**	4.7	11.1	N	1.0	2.8
**G**	5.5	10.4	*	<0.1	14.1
**H**	1.1	4.8	**	<0.1	8.9

BPEAnit in Ethanol Reaction with Ozone

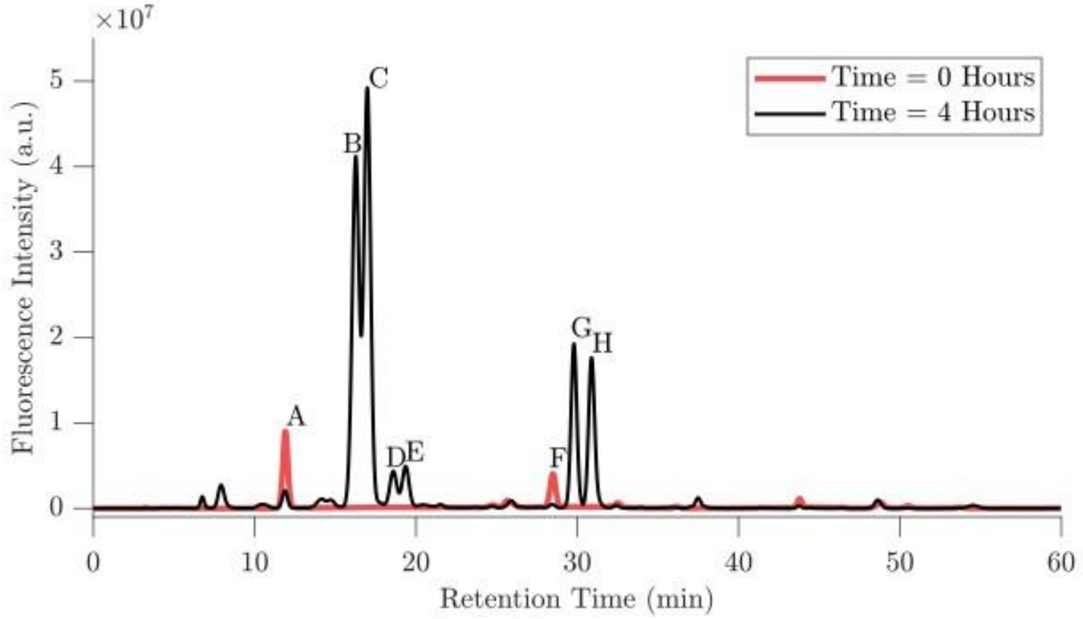

Supplementary Figure 25: Fluorescence HPLC-MS trace (λ_{ex} = 434 nm, λ_{em} = 492 nm) of 0.5 mM BPEAnit in ethanol after 4 h exposure to a constant stream of ozone (ca. 9.5 ppm). BPEAnit is Peak F.

Supplementary Table 4: LC-MS results for the reaction of 0.5 mM BPEAnit in ethanol after 4 h exposure to a constant stream of ozone (ca. 9.5 ppm). BPEAnit is Peak F.

Peak	Retention Time (min)	Observed m/z	Closest Assigned Molecular Formula	Δm/z (ppm)
A	11.9	501.3420	$C_{37}H_{43}N$ (501.3396)	4.79
B	16.3	461.1902	$C_{35}H_{25}O$ (461.1905)	0.65
C	17.0	539.2093	$C_{36}H_{27}NO_4$ (539.2097)	0.74
D	18.6	-[a]	-	-
E	19.4	-[a]	-	-
**F**	28.5	490.2171	**$C_{36}H_{28}NO$ (490.2165)**	1.22
G	29.8	515.2481	$C_{35}H_{33}NO_3$ (515.2460)	4.08
H	30.9	476.2135	$C_{36}H_{28}O$ (476.2140)	1.05

[a] No predominant ion observed.

BPEAnit in Cyclohexane Reaction with Ozone

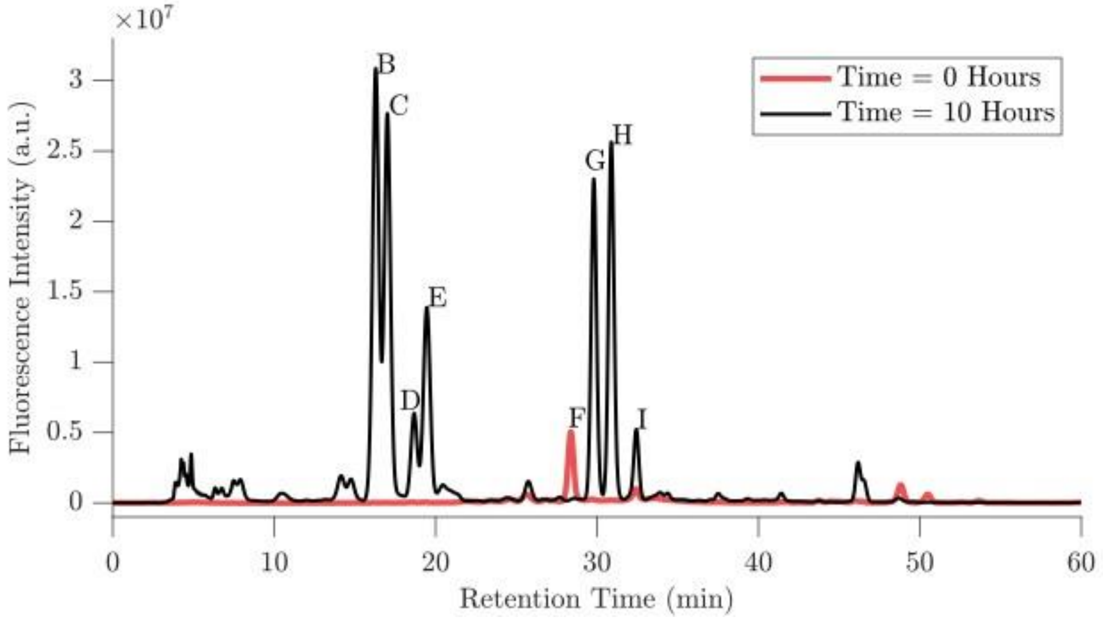

Supplementary Figure 26: Fluorescence HPLC-MS trace (λ_{ex} = 434 nm, λ_{em} = 492 nm) of 0.5 mM BPEAnit in cyclohexane after 10 h exposure to a constant stream of ozone (ca. 9.5 ppm). BPEAnit is Peak F.

Supplementary Table 5: LC-MS results for the reaction of 0.5 mM BPEAnit in cyclohexane after 10 h exposure to a constant stream of ozone (ca. 9.5 ppm). BPEAnit is Peak F.

Peak	Retention Time	Observed m/z	Closest Assigned Molecular Formula	Δm/z (ppm)
B	16.29	461.1902	$C_{35}H_{25}O$ (461.1905)	0.65
C	17.01	539.2093	$C_{36}H_{27}NO_4$ (539.2097)	0.74
D	18.67	-[a]	-	
E	19.45	-[a]	-	
**F**	28.59	490.2168	**$C_{36}H_{28}NO$ (490.2165)**	0.61
G	29.80	515.2481	$C_{35}H_{33}NO_3$ (515.2460)	4.08
H	30.88	476.2135	$C_{36}H_{28}O$ (476.2140)	1.05
I	32.43	519.2195	$C_{37}H_{29}NO_2$ (519.2198)	0.77

[a] No predominant ion observed.
